# The fungal ribonuclease-like effector protein CSEP0064/BEC1054 represses plant immunity and interferes with degradation of host ribosomal RNA

**DOI:** 10.1371/journal.ppat.1007620

**Published:** 2019-03-11

**Authors:** Helen G. Pennington, Rhian Jones, Seomun Kwon, Giulia Bonciani, Hannah Thieron, Thomas Chandler, Peggy Luong, Sian Natasha Morgan, Michal Przydacz, Tolga Bozkurt, Sarah Bowden, Melanie Craze, Emma J. Wallington, James Garnett, Mark Kwaaitaal, Ralph Panstruga, Ernesto Cota, Pietro D. Spanu

**Affiliations:** 1 Department of Life Sciences, Imperial College London, London, United Kingdom; 2 Architecture et Fonction des Macromolécules Biologiques, Aix-Marseille Université, CNRS, Marseille, France; 3 Institute for Biology I, Unit of Plant Molecular Cell Biology, RWTH Aachen University, Aachen, Germany; 4 The John Bingham Laboratory, NIAB, Cambridge, United Kingdom; 5 Centre for Host-Microbiome, Kings College London, London, United Kingdom; CSIRO, AUSTRALIA

## Abstract

The biotrophic fungal pathogen *Blumeria graminis* causes the powdery mildew disease of cereals and grasses. We present the first crystal structure of a *B*. *graminis* effector of pathogenicity (CSEP0064/BEC1054), demonstrating it has a ribonuclease (RNase)-like fold. This effector is part of a group of RNase-like proteins (termed RALPHs) which comprise the largest set of secreted effector candidates within the *B*. *graminis* genomes. Their exceptional abundance suggests they play crucial functions during pathogenesis. We show that transgenic expression of RALPH CSEP0064/BEC1054 increases susceptibility to infection in both monocotyledonous and dicotyledonous plants. CSEP0064/BEC1054 interacts *in planta* with the pathogenesis-related protein PR10. The effector protein associates with total RNA and weakly with DNA. Methyl jasmonate (MeJA) levels modulate susceptibility to aniline-induced host RNA fragmentation. *In planta* expression of CSEP0064/BEC1054 reduces the formation of this RNA fragment. We propose CSEP0064/BEC1054 is a pseudoenzyme that binds to host ribosomes, thereby inhibiting the action of plant ribosome-inactivating proteins (RIPs) that would otherwise lead to host cell death, an unviable interaction and demise of the fungus.

## Author summary

Powdery mildews are common plant diseases which affect important crop plants including cereals such as wheat and barley. The fungi that cause this disease are obligate biotrophs: they have an absolute requirement for living host cells which they penetrate with feeding structures called haustoria. These fungi must be highly effective at avoiding immune recognition which would lead to death of the host cell and the pathogen. We assume they do this by delivering effector proteins to the host. While several hundred secreted effectors have been described in cereal powdery mildews, it is unknown how they work. Here, we use X-ray crystallography and nuclear magnetic resonance (NMR) spectroscopy to determine the structure and interactions of the effector CSEP0064/BEC1054, representative of the largest class of effectors resembling fungal RNases. We find that this effector binds nucleic acids. Expression of the effector in plants increases susceptibility to infection. Moreover, transgenic CSEP0064/BEC1054 expression in wheat inhibits the degradation of host ribosomal RNA induced by ribosome-inactivating proteins (RIPs). We propose a novel mechanism of action for the RNase-like effectors in powdery mildews: they may act as pseudoenzymes to inhibit the host RIPs, known components of plant immune responses that lead to host cell death.

## Introduction

The obligate biotrophic fungus *Blumeria graminis* causes powdery mildew disease on some small grain cereals and grasses (Poaceae). A high degree of host specificity is displayed by the fungus, with at least eight *formae speciales* (f.sp.), each infecting a different host genus. *Blumeria graminis* f.sp. *hordei* and *B*. *graminis* f.sp. *tritici* colonise barley (*Hordeum vulgare* L.) and wheat (*Triticum aestivum* L.), respectively, and they can result in large crop losses [1]. In the case of powdery mildews, the only part of the fungus that actually penetrates the plant during infection is the haustorium [2], a dedicated fungal infection structure thought to absorb plant nutrients. The success of infection is determined by the outcome of a “secretory warfare” between the host and the pathogen, which produces effectors at the haustorial complex [2, 3].

Genomes of powdery mildew fungi code for several hundred effector-like proteins: 491 Candidate Secreted Effector Proteins (CSEPs) were initially identified in the *B*. *graminis* f.sp. *hordei* genome [4], and it has become clear that there are even more in this f.sp. [5], while 844 are currently described in the recently updated *B*. *graminis* f.sp. *tritici* genome [6]. These putative effectors have a predicted amino-terminal signal peptide for secretion, lack recognisable transmembrane domains, and have no relevant BLAST hits outside of the powdery mildew family (Erysiphaceae). As for most other pathogen effectors, their mode of action is not yet understood. Previously, two complementary bioinformatic procedures were used to identify CSEPs with predicted RNA-binding or RNase folds: InterProScan combined with Gene Ontology characterisation, and IntFOLD, an integrated structure prediction server. In total, 72 out of the then 491 known *B*. *graminis* f.sp. *hordei* CSEPs were found by these approaches, with 54 predicted by InterProScan and 37 by IntFOLD (19 of which were found by both techniques [4]). Proteins occurring within CSEP families with structural similarity to RNase and/or RNA-binding activity were termed RNase Like Proteins expressed in Haustoria (RALPHs) [7]. Some of the RALPH CSEP families contain members for which the RNase domain is not recognised by the current prediction algorithms. If the latter are included, the RALPHs comprise the biggest subset of effector candidates within the *Blumeria graminis* f.sp. *hordei* genome, numbering at least 113 of the previously analysed 491 CSEPs. Their abundance, and their proliferation within a genome that otherwise has lost numerous genes [8], suggests that they play a prominent role during infection [4]. This notion is further supported by the fact that some RALPH effectors are recognised by dedicated plant immune receptors and thus act as avirulence factors in the plant-powdery mildew interaction [9] [10]. The genes encoding RALPHs, and other CSEPs, are often physically closely linked to (retro-)transposable elements, indicating that the duplication of these effectors may have occurred through illegitimate recombination of (retro-)transposon sequences [4]. An RNA interference (RNAi)-based screen for functionally important effector genes in *B*. *graminis* f.sp. *hordei* identified eight genes whose expression is required for full pathogenic development. These included two genes encoding RALPH effectors: CSEP0064/BEC1054 and CSEP0264/BEC1011, both belonging to family 21 of the predicted CSEPs in this powdery mildew pathogen [4, 11].

We have previously found four barley proteins that interact with CSEP0064/BEC1054 by *in vitro* affinity assays followed by liquid chromatography mass spectrometry analysis. These proteins comprise a eukaryotic Elongation Factor 1 gamma (eEF1γ), a Pathogenesis Related Protein 5 (PR5), a Glutathione-S-Transferase (GST) and a Malate Dehydrogenase (MDH). The respective protein-protein interactions were confirmed by subsequent yeast two-hybrid (Y2H) experiments [12]. Other barley proteins bind to CSEP0064/BEC1054 *in vitro* but do not interact in yeast. These are Pathogenesis Related Protein 10 (PR10), a ribosomal 40S subunit protein 16 (40S 16), and a eukaryotic Elongation Factor 1 alpha (eEF1α) [12].

The predicted RNase structure of CSEP0064/BEC1054 was originally determined by *in silico* homology modelling. However, the protein, like other RALPHs, is unlikely to be catalytically active, because it lacks the critical active site residues known to be required for RNase activity [4]. Plants also possess RNases that are proposed to be implicated in defence against pathogens. Examples are the RIPs, which depurinate the sarcin-ricin loop (SRL) in the ribosomal RNA (rRNA) of the large ribosomal subunit [13]. In barley, the jasmonate-induced protein of 60 kDa (JIP60) is a RIP involved in mediating host-induced cell death [14, 15].

Here, we show that heterologous transgenic expression of CSEP0064/BEC1054 in wheat enhances susceptibility to *B*. *graminis* f.sp. *tritici*. Similarly, expression of this protein in *Nicotiana benthamiana* increases the susceptibility to the oomycete pathogen *Peronospora tabacina*, suggesting that CSEP0064/BEC1054 subverts a defence mechanism/ pathway conserved among monocotyledonous and dicotyledonous plant species. To provide mechanistic insights into the function of CSEP0064/BEC1054, we solved the structure of the first RALPH by X-ray crystallography, determining unequivocally a high degree of structural similarity with fungal RNases. Furthermore, we confirm that CSEP0064/BEC1054 interacts with ribonucleic acid, and interferes with methyl-jasmonate induced cleavage of RNA in wheat. Together, these findings provide the first evidence for a RALPH-mediated interference with host RNA metabolism. Given the similarity of the predicted structure of *Blumeria* RALPH proteins to RNases and to the core region of fungal RIPs, we hypothesise that RALPHs may stoichiometrically outcompete host RIPs, thereby inhibiting their cell death-promoting activity and thus act as a pseudoenzyme [16].

## Results

### CSEP0064/BEC1054 increases susceptibility to adapted pathogens

We previously assessed the contribution of CSEP0064/BEC1054 to the interaction of barley and its adapted powdery mildew pathogen, *B*. *graminis* f.sp. *hordei*, by Host-Induced Gene Silencing (HIGS; [11]). In this native context, CSEP0064/BEC1054 contributes significantly to fungal virulence: when the respective gene is silenced by HIGS, the infection success (haustorial index) drops to less than half of the value obtained with a control construct.

To explore the effect of CSEP0064/BEC1054 on interactions with additional pathogens, we generated transgenic bread wheat (*T*. *aestivum*) lines constitutively expressing a codon-modified version of *CSEP0064/BEC1054* lacking the N-terminal signal peptide for secretion (termed *wBEC1054*), which was also used in our previous HIGS experiments [11]. We selected progeny of three homozygous T3 lines, two carrying single expressed copies of the *CSEP0064/BEC1054* transgene (+/+, lines 3.3.7 and 3.3.14) and one null segregant (lacking the transgene), referred to as azygous (-/-, line 3.3.12), which served as a negative control in our study.

We first determined whether expression or potential unintended gene disruption by the transgene affects morphology and/or development of the wheat lines. To this end, we investigated the phenotype of adult T4 plants homozygous for the effector transgene (+/+) or respective azygous controls (-/-) under the same experimental conditions as used for subsequent assays. According to common practice for the phenotypic characterisation of wheat plants [17–20], eleven characteristics were assayed quantitatively: leaf number, maximum height, peduncle (internode 1) and other internode lengths, ear length, subcrown length, fertile tiller number, tiller mass and grain number. The respective values for these parameters from the azygous individuals and the transgenics were indistinguishable (S1 Fig); that is, the presence of transgenic *CSEP0064/BEC1054* does not affect the adult phenotype of wheat, in our experimental conditions.

**Fig 1:**
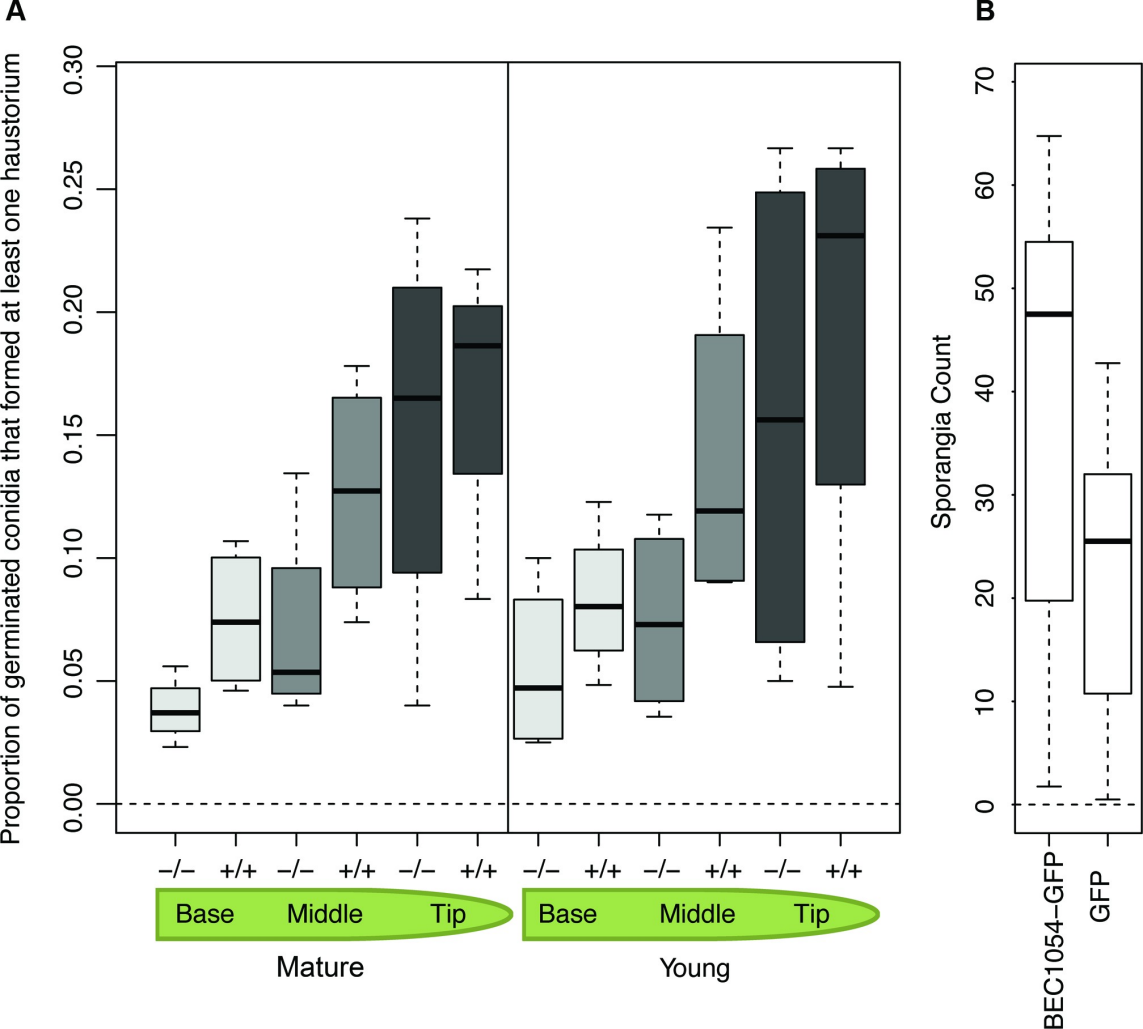
Transgenic expression of CSEP0064/BEC1054 in plants enhances susceptibility to adapted pathogens. **A)** The presence of the transgene *wbec1054*, encoding CSEP0064/BEC1054 from the non-adapted barley powdery mildew pathogen, increases haustorium formation of the adapted pathogen *B*. *graminis* f.sp. *tritici* in wheat. Two centimeter leaf segments were taken from primary leaves (young plants), or from the third most recent leaf (mature plants), and the mean proportion of germinated conidia that produced a functional haustorium (determined by measuring the number of colonies forming an extensive network of epiphytic hyphae) was assessed. Plants were either homozygous (+/+) or azygous (-/-) siblings for *wbec1054*. Young plants were three weeks old, and mature plants were eleven weeks old. The boxes represent the quartiles, the thick line denotes the median, and maximum and minimum values are shown by the error bars. **B)** Sporangia production by the adapted downy mildew pathogen *P*. *tabacina* is increased in *N*. *benthamiana* by expression of CSEP0064/BEC1054 from the non-adapted barley powdery mildew pathogen. *N*. *benthamiana* leaves were infiltrated on one side of the midrib with Agrobacteria delivering plasmids encoding GFP and RFP, and on the other side with Agrobacteria delivering plasmids encoding CSEP0064/BEC1054 with a C-terminal GFP tag and RFP (as a transformation marker). Leaves were inoculated, within one hour of infiltration, with *P*. *tabacina* sporangia on their abaxial surface. After ten days, leaf disks were collected from each leaf, the sporangia removed via washing, counted, and used to calculate the mean. Significantly more sporangia were found with CSEP0064/BEC1054-GFP than with GFP only (n = 5, p<0.001). The thick line denotes the median of each boxplot, the boxes represent the quartiles, maximum and minimum values are shown by the error bars.

We next measured the effect of the *CSEP0064/BEC1054* transgene on the susceptibility of the wheat lines to its adapted powdery mildew pathogen, *B*. *graminis* f.sp. *tritici*. Microcolonies with epiphytic hyphae formed can be used as a proxy for the presence of functional haustoria. This parameter can be expressed as the proportion of microcolonies relative to the number of germinated conidia (propH; Fig 1A). We determined the propH value at the base, the middle and the tip of both young and mature leaves of T4 individuals of the transgenic lines (+/+) and the azygous plants (-/-). The median propH was consistently higher in a statistically significant manner in leaf blades of plants derived from the transgenic lines (+/+) as compared to the controls (-/-), irrespective of the position within the leaf (base, middle, tip) or the age of the leaf (young, mature; Fig 1A; S1 Table). Notably, the spread of the data increased from the leaf base to the leaf tip in both young and mature leaves, as shown by the increasing size of the boxes in the boxplots and their respective error bars (Fig 1A).

To test the contribution of CSEP0064/BEC1054 to a plant-microbe interaction with a dicotyledonous host species and an adapted pathogen different from powdery mildew, we opted for transient expression of *CSEP0064/BEC1054* in *Nicotiana benthamiana* and subsequent challenge with the oomycete pathogen *Peronospora tabacina*, the causal agent of the tobacco downy mildew disease. *Agrobacterium tumefaciens*-mediated transformation was used to transiently express either Green Fluorescent Protein (GFP)-tagged CSEP0064/BEC1054 (C terminal tag), or GFP alone as a control, in *N*. *benthamiana* (in each case co-expressed with RFP as a transformation marker). Leaves from four-week-old leaves were detached, and co-infiltrated with Agrobacteria delivering plasmids encoding CSEP0064/BEC1054-GFP and RFP on one side of the midrib, and with Agrobacteria delivering plasmids encoding GFP and RFP on the other. The leaves were then inoculated with *P*. *tabacina* sporangia on the abaxial surface, and the number of sporangia produced on the leaf after ten days was assayed. The presence of GFP-tagged CSEP0064/BEC1054 significantly increased the mean of the sporangia produced as compared to the GFP control (Fig 1B).

In conclusion, transgenic expression of CSEP0064/BEC1054 promotes virulence of diverse adapted pathogens (*B*. *graminis* f.sp. *tritici*, *P*. *tabacina*) in monocotyledonous (wheat) and dicotyledonous (*N*. *benthamiana*) plant species.

### CSEP0064/BEC1054 interacts with PR10 *in planta*

**Fig 2:**
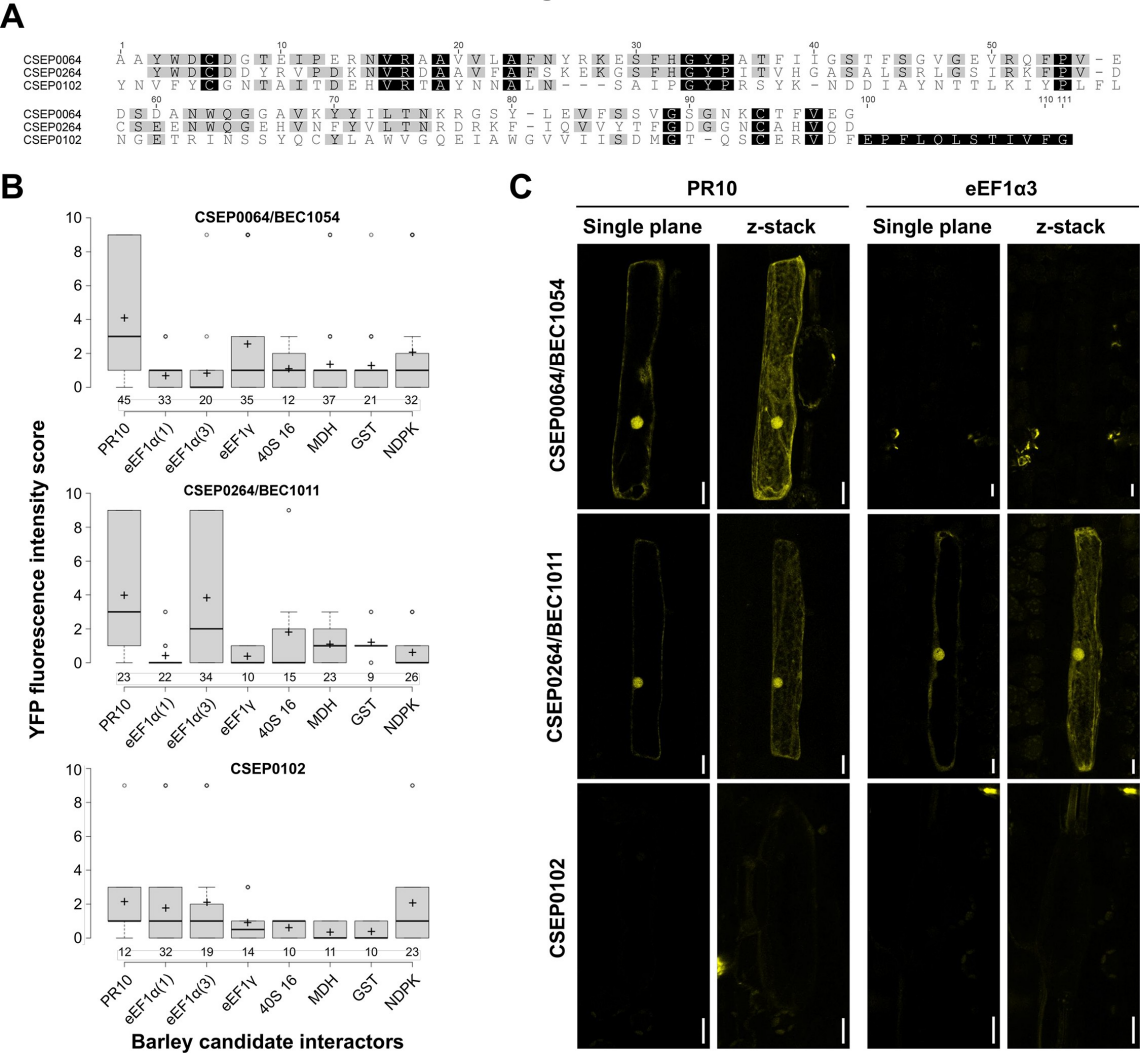
CSEP0064/BEC1054 interacts with barley PR10 *in planta*. A) Multiple amino acid sequence alignment of CSEP0064/BEC1054, CSEP0264/BEC1011 and CSEP0102. The alignment was generated by CLUSTALW using the mature polypeptides (i.e., the predicted N-terminal signal peptides removed). Amino acids that are identical between all three proteins are highlighted in black, amino acids shared by two of the three proteins are shown in gray. B) The host plant, barley, was used to perform a BiFC assay via particle bombardment-mediated transient gene expression in single leaf epidermal cells. As bait proteins, CSEP0064/BEC1054, CSEP0264/BEC1011 and CSEP0102 were used. The barley prey proteins were: Pathogenesis-Related protein 10 (PR10), eukaryotic Elongation Factor 1 Gamma (eEF1γ), eukaryotic Elongation Factor 1 Alpha (eEF1α) isoforms 1 and 3, malate dehydrogenase (MDH), Glutathione-S-Transferase (GST) and nucleoside diphosphate kinase (NDPK; negative control). BiFC signals were scored semiquantitatively by assigning transformed (DsRED-expressing) cells manually to one of four categories based on YFP fluorescence intensity. Center lines of box plots show the medians of the scores; box limits indicate the 25th and 75th percentiles as determined by R software; whiskers extend 1.5 times the interquartile range from the 25th and 75th percentiles; outliers are represented by dots; crosses designate the arithmetic mean of the scores; and numbers of inspected cells are given below the boxes. C) Representative micrographs of BiFC-positive barley epidermal cells for the combinations CSEP0064/BEC1054-PR10 (upper panel), CSEP0064-PR10 (middle panel) and CSEP0064-eEF1α(3) (lower panel). For each combination a single focal plane and a maximum projection (z-stack) are shown. Scale bars are 20 μM.

In order to test physical *in planta* interactions between CSEP0064/BEC1054 and several host proteins previously identified by protein *in vitro* affinity-LCMS experiments and targeted Y2H assays (PR5, PR10, eEF1α, eEF1γ, ribosomal protein 40S 16, MDH and GST; [12]), we performed BiFC experiments with the fungal effector and the respective candidate targets [21]. We excluded PR5 from this analysis since PR5 is known to represent a secreted protein with extracellular localisation, but included in addition a barley nucleoside-diphosphate kinase (NDPK) that was used as a negative control in previous Y2H assays [12]. Apart from CSEP0064/BEC1054 we added a closely related CSEP (CSEP264/BEC1011, CSEP family 21, 45% amino acid identity to CSEP0064/BEC1054) and a more distantly related CSEP (CSEP0102, CSEP family 20, 17% amino acid identity to CSEP0064/BEC1054) as bait proteins to these experiments (Fig 2A). Similar to CSEP0064/BEC1054, both CSEP0264/BEC1011 and CSEP0102 are predicted to be RNase-like proteins.

We first analysed the *in planta* subcellular localisation of all putative interaction partners (three CSEP bait and eight barley prey proteins). The bait and prey proteins were translationally fused at the C-terminus with monomeric yellow fluorescent protein (mYFP) and transiently co-expressed with the red-fluorescent protein DsRED (as a transformation marker) in barley leaf epidermal cells [22]. Expression and subcellular localisation were determined via confocal microscopy (S2 Fig). In the case of all CSEPs, there was a weak and diffuse fluorescence signal in the cytoplasm and the nucleus; in addition, occasionally a punctate distribution of these proteins was observed in the cytoplasm. The PR10-mYFP fusion was distributed fairly homogeneously throughout the cytoplasm, with brighter fluorescence evident in the nucleus. The GST-mYFP fusion protein yielded a very diffuse and faint signal in the cytoplasm and nucleus, with bright puncta in the cytoplasm, and a bright signal in a section of the nucleus. Fluorescence of three elongation factor fusions (eEF1γ-mYFP, eEF1α(1)-mYFP and eEF1α(3)-mYFP) was detectable in the cytoplasm, and for eEF1α(1)-mYFP and eEF1α(3)-mYFP (two paralogs of eEF1α we cloned from barley) fluorescence was also observable in the nucleus. The 40S 16-mYFP fusion protein yielded a very diffuse and faint signal in the cytoplasm and the nucleus, with bright puncta in the cytoplasm, and a bright signal in the nucleus. The NDPK- and MDH-mYFP fusions were both observed in the nucleus and cytoplasm (S2 Fig). Taken together, all bait and prey fusion proteins yielded detectable *in planta* mYFP fluorescence and were present in the cytoplasm and in part in the nucleus, allowing for potential interaction in these cellular compartments.

BiFC analysis was performed in barley leaf epidermal cells by co-expression of the three CSEP proteins (CSEP0064/BEC1054, CSEP0264/BEC1011 and CSEP0102), N-terminally tagged with the C-terminal half of mYFP, the barley prey proteins C-terminally tagged with the N-terminal domain of mYFP, and DsRED (as a transformation marker). We semiquantitatively assessed BiFC-mediated YFP fluorescence intensity based on 9–45 inspected cells per tested bait-prey combination and four distinct phenotypic categories (see Materials and Methods for details). Overall, we noticed some cell-to-cell variation of the BiFC signal, consistent with the known propensity of the BiFC assay to stabilise weak protein-protein associations depending on experimental conditions and expression levels [23]. Nonetheless, for CSEP0064/BEC1054, a high number of cells with a clear YFP signal was found upon co-expression with PR10 (Fig 2B and 2C). This situation differed from the six other tested barley candidate proteins and the NDPK negative control, for which only occasionally fluorescent cells, typically with lower fluorescence intensity, were found (Fig 2B). For CSEP0264/BEC1011, which is closely related to CSEP0064/BEC1054 (45% amino acid identity; Fig 2A), we likewise scored a positive interaction with PR10. In addition, the CSEP0264/eEF1α(3) combination yielded a high number of fluorescent cells (Fig 2B and 2C). By contrast, all tested interactions of barley prey proteins with CSEP0102, which is more distantly related to CSEP0064/BEC1054 (17% amino acid identity; Fig 2A), failed to show a consistent BiFC signal (Fig 2B). In case of the positive bait-prey pairings, YFP fluorescence was observed in the nucleus and in the cytoplasm. This pattern largely matched the subcellular localisation of the CSEP bait proteins expressed alone (S2 Fig).

### CSEP0064/BEC1054 shows structural similarity to T1 RNases

To provide detailed insights into the molecular architecture of RALPH effectors, we determined the structure of CSEP0064/BEC1054 by X-ray crystallography. An initial model obtained from a dataset phased by iodide-SAD (single-wavelength anomalous diffraction) was subsequently used to calculate phases for a native dataset at 1.3 Å via molecular replacement. Data processing and refinement statistics for the structures are outlined in S2 Table.

**Fig 3:**
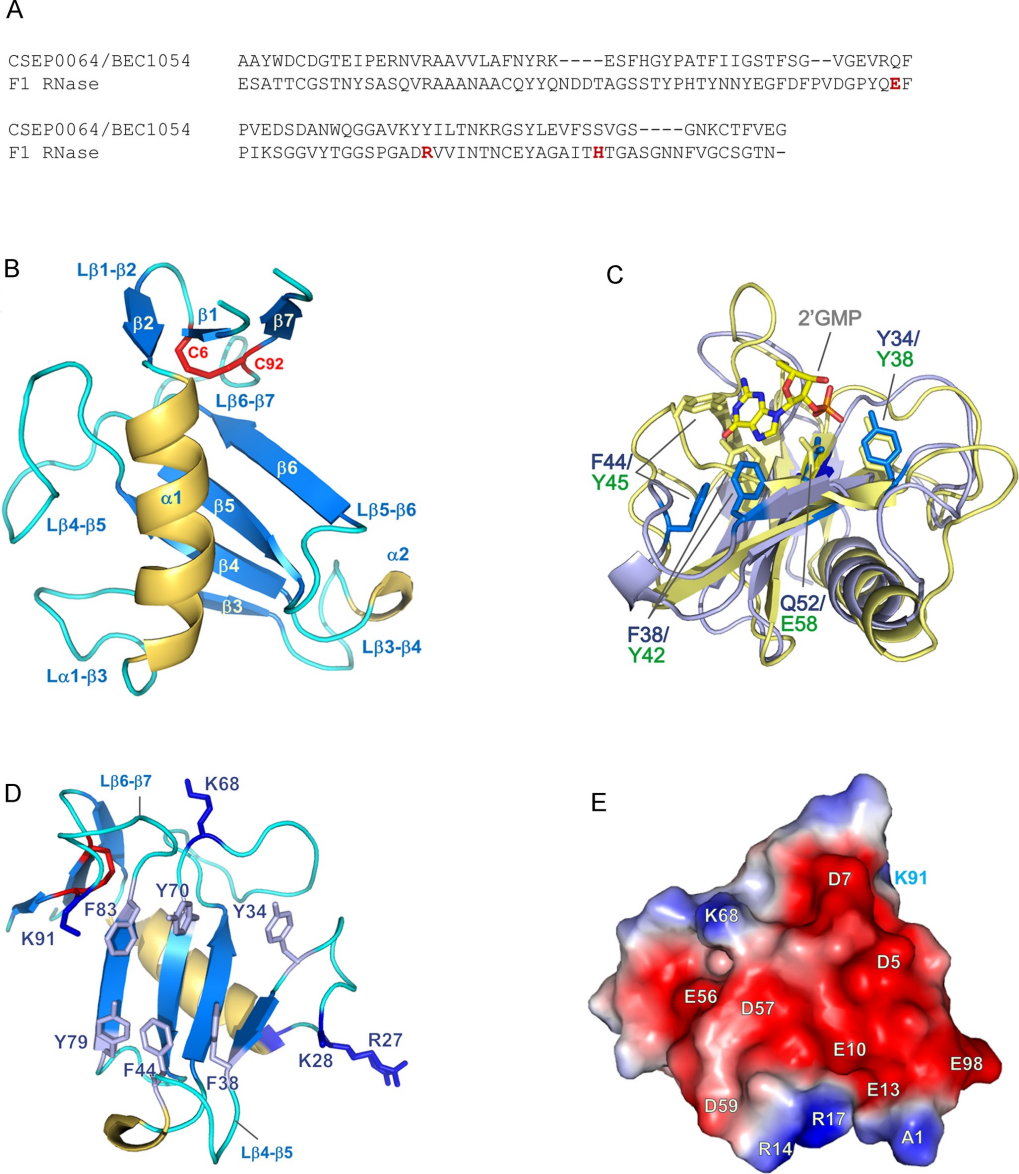
Structural details of CSEP0064/BEC1054. **(A)** Structure-based sequence alignment of CSEP0064/BEC1054 and the F1 RNase from *F. moniliforme*. Residues forming the catalytic triad in the F1 RNase are marked in red. **(B)** CSEP0064/BEC1054 (PDB ID 6FMB) exhibits a canonical T1 RNase type fold, including a disulphide bridge between C6 and C92 linking the N- and C- termini of the protein (shown in red). **(C)** Structural overlay of CSEP0064/BEC1054 (backbone in light blue) with F1 RNase from *F*. *moniliforme* (backbone in yellow, PDB ID 1FUT). These structures share an RMSD of 2.3 Å over 64 Cα atoms. Residues of the F1 RNase that bind 2’GMP are highlighted in yellow and functionally equivalent residues in non-catalytic CSEP0064/BEC1054 in marine blue. For clarity, the side chains of Y70 (in CSEP0064/BEC1054) and R77 (in F1 RNase) are not shown. **(D)** Exposed aromatic side chains in the large β-sheet and the β1-β3, β3-β4 loops cluster on the concave face of the CSEP0064/BEC1054 protein. Side chains of positively charged side chains at the edges of the concave face are also shown. **(E)** Electrostatic surface potential calculated using APBS [30], shows a patch (shown in red) of negatively charged residues on the surface of CSEP0064/BEC1054.

Analysis using the Dali server [24] confirmed that CSEP0064/BEC1054 is a close structural homologue of T1 RNases, a family of enzymes with high degree of specificity for cleavage of substrates at the 3’ end of guanosyl residues. The crystal structure reveals a canonical (α+β) fold comprising a core four-stranded anti-parallel β-sheet packed against an α-helix, and a second shorter peripheral anti-parallel β-sheet formed from the N-terminal hairpin and C-terminal strand β7 (Fig 3A). This arrangement is constrained by a disulphide bridge formed between residues C6 and C92, a feature that is universally conserved in the T1 RNase family [25].

**Fig 4:**
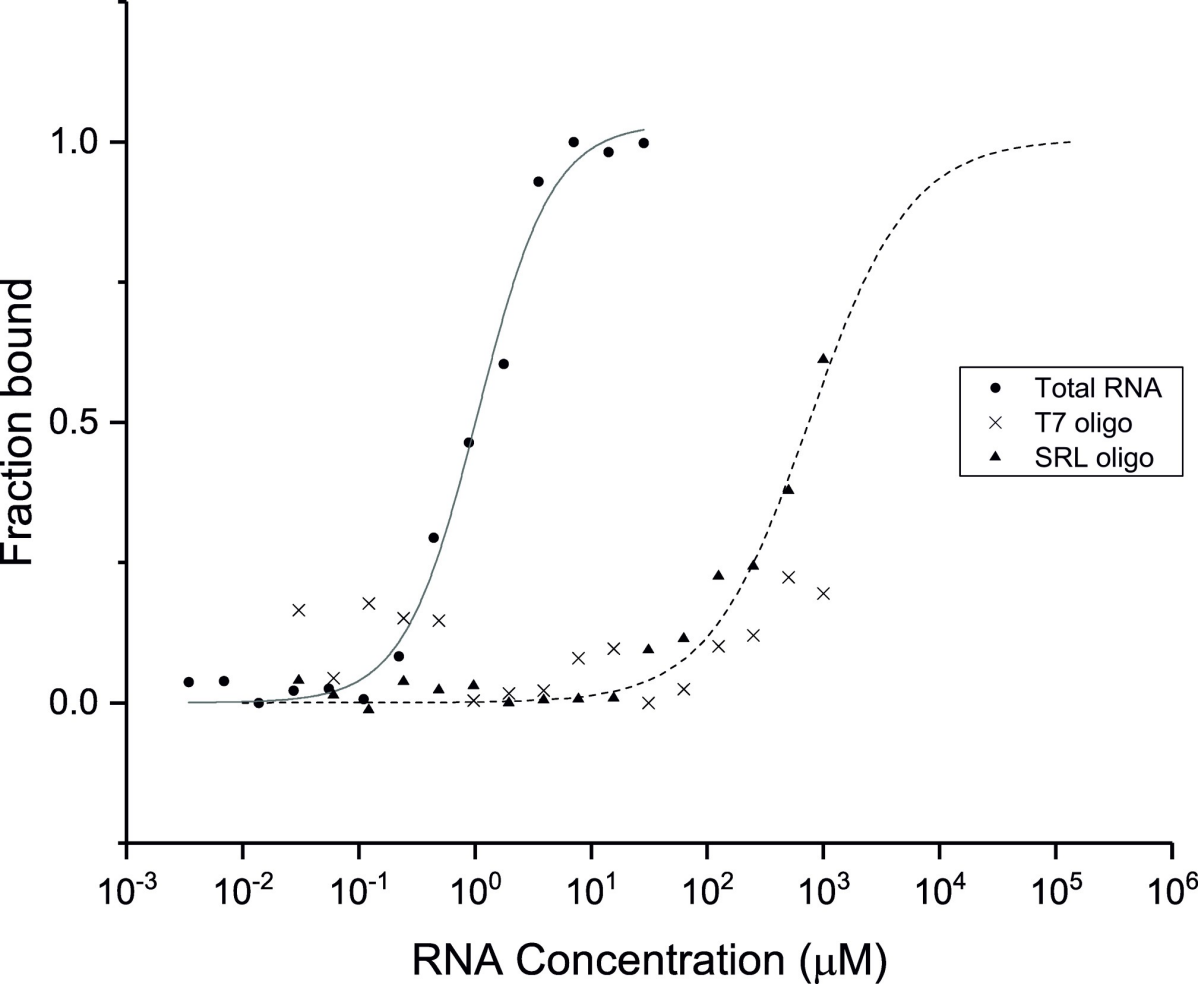
CSEP0064/BEC1054 binds total RNA. Isotherms from microscale thermophoresis assays to determine the *in vitro* RNA-binding capacity of recombinant CSEP0064/BEC1054 protein. Tested RNA species were barley total RNA (●, K_D_ = 1.0±0.1 mM), a ribosomal SRL RNA sequence (▲, K_D_ = 675±52 mM) and a bacteriophage T7 RNA promoter sequence (X). Curves shown are the average of two experimental replicates. In the case of the bacteriophage T7 RNA promoter sequence (X), no curve could be fitted.

In T1 RNases, a catalytic triad is located inside a concave face formed by the main β-sheet and loops β3-β4/β6-β7 [26, 27]. Notably, this triad is absent in CSEP0064/BEC1054 (Fig 3B,[4]), but several other features in the protein suggest a role in nucleic acid binding. Structural superposition of CSEP0064/BEC1054 with *F*. *moniliforme* F1 RNase (1FUS), indicates that contacts made by the RNase with the phosphate and ribose moieties of 2’ guanosine monophosphate (2’GMP) are conserved (Fig 3C). Residues Y34 and Q52 in CSEP0064/BEC1054 overlay with residues Y38 and E58 from the F1 RNase that form contacts with the phosphate moiety of 2’GMP. Additionally, catalytic residue R77 is replaced by Y70 in CSEP0064/BEC1054, a residue that may form polar contacts with the phosphate.

Differences in the conformation of loops β3-β4 and β6-β7 account for the main variations in the active site architectures between CSEP0064/BEC1054 and other T1 RNases. In the crystal structures of T1 RNases, these loops form an occluded binding cleft for the substrate [27–29]. In the crystallized conformation of CSEP0064/BEC1054, these loops do not fold over, leaving the main β-sheet with a larger exposed hydrophobic surface (Fig 3D). Relative to the T1 RNases, CSEP0064/BEC1054 has a more compact β3-β4 loop, as it is two residues shorter and contains a single-turn 3–10 α-helix. In this loop, Y42 and Y45 of the F1 RNase are involved in π stacking interactions with the guanosine base [28]. Reconstruction of a comparable active site geometry would require displacement of F38 and F44 in the loop upon the concave surface of CSEP0064/BEC1054 (Fig 3D). Additionally, residues N43 and N44 in F1 RNase establish hydrogen bonds with guanosine base substrates via backbone atoms; a conformational change has been observed for these residues between ligand-free and 2’GMP-bound forms of the protein [28]. For a comparable interaction, a movement in the same loop would be required to bring the backbone atoms of residues I39 and I40 in CSEP0064/BEC1054 within hydrogen bonding distance of the guanosine base.

Charged amino acid residues are clearly segregated on the protein surface (Fig 3E). Most of the negatively charged side chains cluster in the vicinity of a small β-sheet: strands β1, β2 and β7 (residues D5, D7, E10, E13, E56, D57, D59, E81 and E98), whereas the majority of positively charged residues localises to the periphery of the concave surface (R27, K28, K68 and K91, Fig 3D). In T1 RNases, these positions flank the RNA binding site, and it is thus feasible that these residues in CSEP0064/BEC1054 interact with the phosphate backbone of nucleic acids. Intriguingly, the structures of RNases T1 from *Aspergillus oryzae* (9RNT) [27] and F1 from *F*. *moniliforme* (1FUS) [28] do not display the same segregation of positive and negative charges on the protein surface.

The concave surface of CSEP0064/BEC1054 is lined with aromatic residues (Y34, F38, F44, Y70, Y79 and F83) that could be involved in base-stacking interactions with nucleic acids (Fig 3D). Alternatively, these residues could contain a binding site for host proteins, resembling the conserved hydrophobic patches of effectors AVR1-CO39 and AVR-Pia from the rice blast fungus *Magnaporthe oryzae* [30].

### CSEP0064/BEC1054 interacts with RNA *in vitro*

Due to the structural similarity between CSEP0064/BEC1054 and other fungal RNA-binding proteins, we tested potential association of the protein with RNA. Firstly, CSEP0064/BEC1054 was labelled with the fluorophore NT-927 and titrated against increasing concentrations of total RNA extracted from barley up to a final concentration of 10 μg/μl in microscale thermophoresis (MST) experiments (Fig 4). An interaction with total RNA was observed, and whilst the complex nature of the substrate precludes accurate calculation of a K_D_, we estimate this to be in the low micromolar range based on an averaged molecular weight for the most abundant RNA species (rRNA) within a pool of extracted total RNA.

To further probe interactions with specific rRNA sequences, the protein was titrated with an RNA oligonucleotide corresponding to specific 32 bp sequence derived from the SRL of the barley 28S rRNA (Fig 4B). Affinity for this substrate was over two orders of magnitude weaker than for barley total RNA and the interaction could not be saturated with up to 1 mM ligand using MST, preventing a precise estimation of a K_D_. Isotherms suggest that the interaction affinity (K_D_) is likely to be in the high μM-low mM range (Fig 4), suggesting that this rRNA region may not be the biologically relevant ligand for CSEP0064/BEC1054. However, no binding was observed to an RNA oligonucleotide with the sequence of the bacteriophage T7 promoter (a non-rRNA control) under the same conditions, suggesting that CSEP0064/BEC1054 exhibits some binding specificity to ribosomal ribonucleic acids (Fig 4). No further evidence for sequence specificity was observed.

### NMR solution studies highlight regions of conformational flexibility involved in ligand binding

CSEP0064/BEC1054 was further structurally characterised in solution via NMR spectroscopy following isotopic ^15^N and ^13^C labelling of the protein. S3 Fig shows the ^1^H-^15^N HSQC spectrum for CSEP0064/BEC1054, where each cross peak corresponds to an amide group in the protein. Good dispersion of cross peaks indicates that the protein is monomeric and well-folded in solution. Using conventional 3D experiments (see Methods), backbone resonances from 89 out of 94 residues were assigned (i.e. excluding the backbone imines of three prolines). Unassigned residues also display large crystallographic B factors (S42, F44, G46, G64 and S88), indicating that they experience motions that prevent sampling of a defined chemical environment (e.g. at intermediate exchange regime in NMR timescales). These residues map to loop regions on opposite faces of the core β sheet, which suggests that ligand binding by CSEP0064/BEC1054 may involve displacement of these regions towards a structure that resembles the ligand bound conformation of T1 RNases.

**Fig 5:**
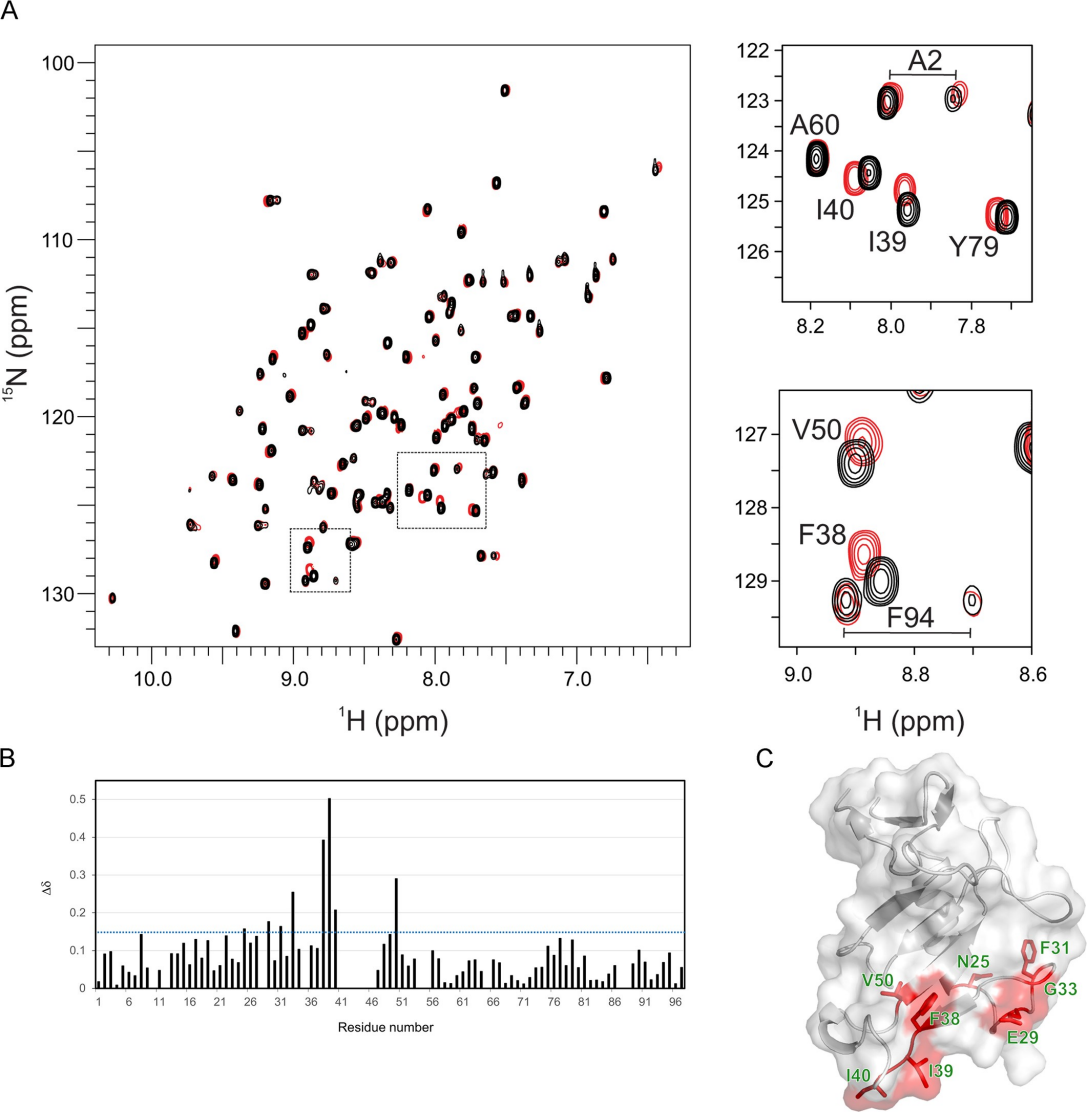
CSEP0064/BEC1054 exhibits discrete chemical shift perturbations that map to its putative RNA-binding site/face. **A)** Overlay of ^1^H-^15^N HSQC NMR spectra of ^15^N-labelled CSEP0064/BEC1054 in the presence (red) and absence (black) of a 50x molar excess of a DNA encoding the sequence of the SRL sequence. The largest chemical shift perturbations (CSPs) are highlighted in the spectrum and enlarged in separate panels on the right. **B)** Plot of chemical shift perturbations (Δδ) of CSEP0064/BEC1054 upon addition of SRL DNA. For each residue, plotted CSPs are the sum of normalised shifts (ΔδN + 5x ΔδH) [31]. No information was available for prolines and other twelve residues that were overlapped with other peaks during titration (E10, T43, V55, Q53), with weak NMR signals or unassigned (notably residues 41–46 in the β3-β4 loop). **C)** Residues that display the most significant CSPs upon ligand binding (with a Δδ larger than 0.15 in **B**) these are mapped onto the crystal structure of CSEP0064/BEC1054.

To provide further insights into the nature of these interactions and the mechanism of binding, chemical shift perturbation (CSP) analyses using ^1^H-^15^N HSQC NMR spectra were analysed upon titration of CSEP0064/BEC1054 with the barley SRL RNA sequence. Titration with 8 mole equivalents of this ligand induced discrete chemical shift changes in a subset of resonances. To reach saturation of CSEP0064/BEC1054, the titration was repeated with a single-stranded SRL DNA oligonucleotide predicted to have the same fold as the RNA SRL oligonucleotide (it was not possible to obtain enough single-stranded RNA, for this purpose). This induced small CSPs for the same residues observed in the RNA SRL titration, which showed saturation at ~50 molar equivalents (Fig 5A). The continuous shift in the position of resonances upon titration indicated that binding was weak, with a K_D_ above 10 μM (i.e. in fast chemical exchange in NMR timescales), thus correlating with the results obtained from MST. In these experiments, solution conditions were carefully controlled after each titration to ensure that the observed CSPs were not an artefact from pH changes.

CSPs were mapped onto the crystal structure of CSEP0064/BEC1054, (Fig 5B and 5C). Interestingly, residues with the largest chemical shifts are F38 (β3), I39, I40 (loop β3-β4) and V50 (β4), i.e. in the region containing residues that determine ligand binding specificity in T1 RNases, as described above. A second subset of CSPs (N25, E29, F31, G33) map in the loop α1-β3, where ligand binding is also expected to induce changes in the chemical environment of amides after interaction with the neighboring β3-β4 loop.

### CSEP0064/BEC1054 inhibits MeJA-dependent degradation of host rRNA

**Fig 6:**
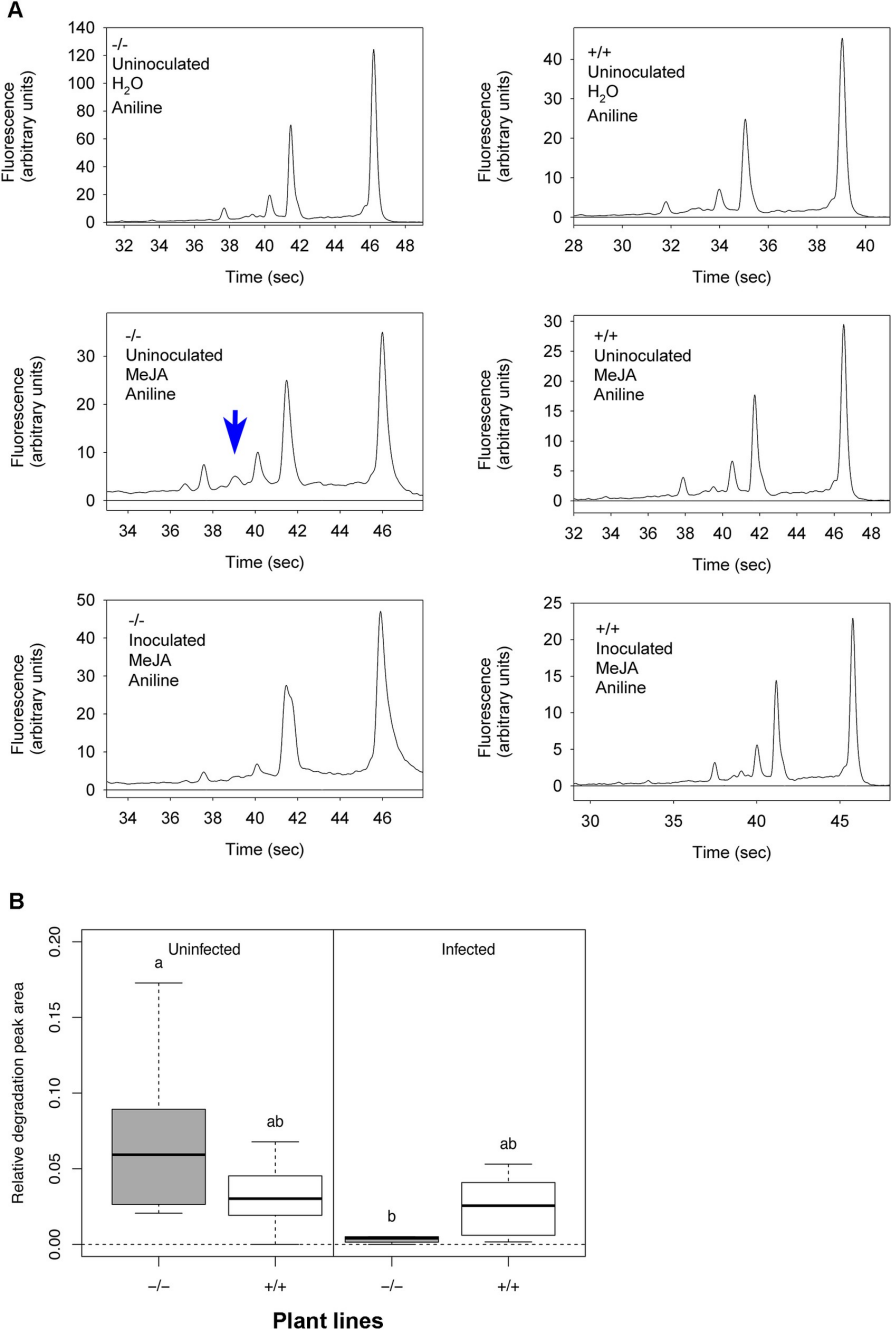
Transgenic expression of CSEP0064/BEC1054 inhibits specific MeJA-dependent aniline-induced rRNA degradation. **A)** Electropherogram peak analysis of total RNA from wheat leaves. Seven day-old transgenic wheat seedlings, either homozygous (+/+) or azygous (-/-) for *wbec1054*, encoding CSEP0064/BEC1054, were inoculated once a day with *B*. *graminis* f.sp. *tritici* for three days or maintained uninoculated. Primary leaves were harvested, and floated on either water or 40 μM MeJA for a further five days. Following total RNA extraction, RNA was treated with aniline, and then examined using an Agilent Bioanalyzer. The blue arrow indicates the RNA degradation peak of interest, which appears after treatment of the total RNA with aniline. **B)** Quantitative analysis of the assay results shown in **A**. The area of the degradation peak revealed in **A** was calculated in relation to the area of the 28S rRNA of the large ribosomal subunit. *Post-hoc* statistical tests were used to determine whether homozygous (+/+) and azygous (-/-) plants undergoing the same treatment were significantly different, as is indicated by different letters. The boxes represent the quartiles, the thick line denotes the median, and maximum and minimum values are shown by the error bars.

JIP60 is a RIP ([32]) whose transcript and protein accumulates in response to treatment with MeJA, a stress-related phytohormone in plants [13]. The action of JIP60 on rRNA in plants can be observed by the accumulation of a characteristic degradation product, visible as new RNA species following *in vitro* treatment of total RNA with aniline [33]. We incubated primary leaves of the transgenic wheat plants constitutively expressing CSEP0064/BEC1054 with MeJA, extracted total RNA, and treated it with aniline. We then analysed the respective RNA fragments by separation in a microchannel-based electrophoretic cell. In the RNA samples obtained from leaves of azygous wheat (negative controls), the aniline treatment resulted in the appearance of a small peak that migrated at an apparent size of about 1,200 bases (Fig 6, S6 Fig). In the RNA profiles from CSEP0064/BEC1054 transgenic leaves processed in the same way, the area of this peak was reduced. Likewise, this peak was reduced or absent in RNA from CSEP0064/BEC1054 transgenic plant leaves extracted at eight days post inoculation with *B*. *graminis* f.sp. *tritici* or at five days after MeJa treatment. In the controls with no MeJA induction, the area of the peak was very small or not detectable (Fig 6A). In sum, these data suggest that MeJa treatment triggers fragmentation of rRNA, which is indicated by the occurrence of a presumptive degradation product. This process appeared to be impeded by the presence of CSEP0064/BEC1054 or by powdery mildew infection.

We then measured these effects quantitatively. The area of the peak representing the presumptive rRNA degradation product was determined, relative to the area of the small (18S) and large (28S) rRNAs. The abundance of the newly occurring RNA species was estimated to be up to about 10% of the 28S rRNA peak, depending on the experimental conditions (Fig 6B). The average areas of the peak corresponding to the putative degradation product in RNA from uninfected plants homozygous for the CSEP0064/BEC1054 transgene (line 3.3.14) was lower than in the azygous controls (line 3.3.12), but this difference is not statistically significant. In azygous plants, powdery mildew infection further decreased the formation of the degradation peak, almost completely abolishing it. The infection status had a statistically significant effect on the mean area of the presumptive degradation peak: overall, powdery mildew infection prevented the formation of the peak. The marked decrease in the degradation peak area also occurred in another transgenic line (line 3.3.7; S7 Fig). The statistical analysis of the effect of the transgene showed that the peak area was intermediate between that of the azygous infected and non-infected leaves, and was not significantly different from either. Therefore expression of CSEP0064/BEC1054 was not sufficient to inhibit the action of the MeJA-induced RNA degradation, to the extent seen in infected plants.

## Discussion

### *In planta* expression of CSEP0064/BEC1054 enhances susceptibility to diverse pathogens

CSEP0064/BEC1054 is a barley powdery mildew candidate effector protein that is highly expressed at early stages of infection [34]. Full expression of the gene is necessary for virulence of *B*. *graminis* f.sp. *hordei*. [11]. Here we investigated further the molecular and structural basis for function of this presumptive pathogen effector.

First, we measured the proportion of germinating conidia that lead to the formation of functional haustoria (propH) as deduced by the development of fungal microcolonies on the surfaces of infected leaves on transgenic wheat expressing CSEP0064/BEC1054. PropH was significantly higher in leaves from plants that are homozygous for the transgene, compared to the azygous controls (Fig 1A). We noted differences in the outcome along the leaf blade: the effect of the transgene was more marked at the base of the leaf blades, intermediate in the middle sections and all but disappeared at the apical sections. This is not altogether surprising as leaf maturation in monocots displays a basipetal pattern [35, 36], and gene expression also occurs in a longitudinally non-uniform manner for leaves of species belonging to the Poaceae [37–39]. The slope in propH could be related to the basipetal gradients observed for immune-related transcripts. Genes encoding transcriptional regulators, such as WRKY factors, display differential expression along leaf blades, with two rice genes coding for WRKY proteins being expressed most highly at the leaf tip, and 13 most highly at the leaf base [39]. Several WRKYs play central roles in modulating disease resistance [40]. Therefore, a gradient in WRKYs and other proteins that control disease resistance [39] could explain the differences along the leaf blades in the effectiveness of CSEP0064/BEC1054 in counteracting host defence we observed in our experiments. Similar patterns of susceptibility were also recently observed in barley leaves infected by *P*. *palmivora* [41].

In a separate set of experiments, leaves subjected to *Agrobacterium*–mediated transient expression of CSEP0064/BEC1054 with a C-terminal GFP tag in the dicot *N*. *benthamiana* were subsequently inoculated with the oomycete *P*. *tabacina*. Expression of CSEP0064/BEC1054-GFP led to an increase in the *P*. *tabacina* sporangia formed when compared with a GFP-only control, indicating an increased susceptibility to the oomycete pathogen (Fig 1B).

Taken together, these results point to CSEP0064/BEC1054 affecting plant immunity that limits the infection with pathogens adapted to their specific host. Importantly, the immune pathways targeted by the candidate effector appear to be conserved in monocotyledonous and dicotyledonous plant species, and affect defence against both fungal and oomycete biotrophic pathogens.

### Interaction of CSEP0064/BEC1054 with host protein PR10 and RNA

Several host proteins interact with CSEP0064/BEC1054 *in vitro* and in yeast [12]. Here, we used BiFC to test whether these host targets associate with CSEP0064/BEC1054 in barley epidermal cells, i.e. in the native cell context of this powdery mildew effector candidate. The fluorescence signal of CSEP0064/BEC1054 tagged with intact mYFP, as control, was diffuse and less intense in comparison to the fluorescence of mYFP-tagged candidate interaction partners (S2 Fig). Faint signals could be due to poor translation, misfolding and/or rapid turnover of this fusion protein–processes that might be indicative of intolerance of this protein—at least at high levels—in eukaryotic/host cells. This scenario would be consistent with the cell growth interference phenotype previously observed in yeast expressing CSEP0064/BEC1054 [12].

Of the seven tested proteins, only PR10 interacted reproducibly with CSEP0064/BEC1054 in BiFC experiments in barley (Fig 2B and 2C). The fluorescent signal matched the expression and subcellular localisation pattern observed for CSEP0064/BEC1054-mYFP (S2 Fig). While this interactor was originally identified in affinity-LCMS experiments, the Y2H tests for PR10 had given inconclusive evidence of interaction due to up-regulation of the β-galactosidase reporter gene, and inconsistent results from the other reporter systems used. Apart from CSEP0064/BEC1054, also the closely related CSEP0264 gave positive BiFC signals with PR10. This CSEP in addition interacted with eEF1α(3), while all other tested interactions yielded essentially background signals (Fig 2B). Absence of positive BiFC signals does not necessarily mean that the respective interactions do not occur *in planta*. We only tested one of the four possible BiFC constellations regarding the tagging of the bait and prey proteins with halves of the YFP fluorophore. Further experiments will thus be needed to obtain a more comprehensive picture of the *in planta* host targets of CSEP0064/BEC1054.

The fact that the PR10 host protein is capable of consistently associating with CSEP0064/BEC1054 in independent, orthogonal assays (affinity-LCMS and BiFC) supports the notion that this interaction may reflect the situation in the true powdery mildew (barley–*B*. *graminis* interaction) context. This idea is further supported by the fact that the closely related CSEP0264, but not the distantly related CSEP0102, also interacts with PR10 (Fig 2B and 2C). The existence of multiple interactions between single effectors and host proteins is not unprecedented and has been well documented in several instances [42, 43]. The fact that eEF1α(3), which was originally identified as an interactor of CSEP0064/BEC1054 in *in vitro* and Y2H assays, yielded positive BiFC signals with CSEP0064 but not CSEP0064/BEC1054 may indicate that CSEP0264 is the *bona fide* interactor of this host protein *in planta*. Cross-interaction of eEF1α(3) and CSEP0064/BEC1054 in affinity-LCMS and in yeast could be based on the high sequence similarity of CSEP0064/BEC1054 and CSEP0264 (Fig 2A), which may allow for the association of CSEP0064/BEC1054 with eEF1α(3) in non-native conditions.

The significance of interactions between pathogen effectors and the PR10 protein may be that they are capable of modulating the antimicrobial activity of these defence-related polypeptides. PR10 proteins belong to the family of Bet v 1 (birch pollen allergen) homologs [44]. Interestingly, some PR10 proteins have been reported to have RNase activity [45]. An unrelated barley PR protein (PR17c) that is required for defence against *B*. *graminis* f.sp. *hordei* also physically interacts with a *B*. *graminis* f.sp. *hordei* candidate effector protein (CSEP0055; [46]). These putative effectors may somehow modulate the antifungal activity of the PR proteins. It remains to be seen whether this is indeed the case for the CSEP0064/BEC1054-PR10 interaction.

Our studies also aimed to test whether CSEP0064/BEC1054 binds RNA. Binding isotherms using MST show a clear interaction with total RNA, suggesting that the protein can either bind to RNA unspecifically or that it is capable to recognise a distinct sequence in a well populated species, e.g. an rRNA motif. Experiments using RNA oligonucleotides show that CSEP0064/BEC1054 recognises the SRL of rRNA as a specific ligand, relative to a sequence encoding the bacteriophage T7 promoter, used as a negative control (Fig 5). Using ^1^H-^15^N HSQC NMR spectra, we observed discrete chemical shifts changes upon titration of the SRL RNA oligonucleotide, indicating a weak interaction. This implies that, for a biologically relevant association, the protein may require additional binding elements such as those present in an RNA-protein complex like the intact ribosome, or that a different RNA sequence is recognised *in vivo*.

### Structural similarity of CSEP0064/BEC1054 to RNases and RIPs

Structural modelling of CSEPs from the *B*. *graminis* f.sp. *hordei* genome [4] suggested that RNase-type folds are considerably overrepresented relative to other protein folds. For example, CSEP0064/BEC1054 (from CSEP family 21) was predicted to exhibit a fold characteristic to members of the T1 RNase family. As previously noted [4], *B*. *graminis* f.sp. *hordei* RALPHs lack the canonical catalytic triad of RNases and are thus unlikely to possess RNase activity. Nonetheless, two main structural features indicated that CSEP0064/BEC1054 may be an RNA-binding protein. In fact, (*1*) the distribution of specific positive charges and (*2*) the clustering of aromatic side chains in the concave face of the domain (Fig 3D) are similar to that observed on the surfaces of F1 RNases involved in RNA ligand binding [27, 47]. However, it appears that these features would not confer CSEP0064/BEC1054 the same substrate specificity observed in case of these RNases. In particular, this RALPH candidate effector lacks the specific arrangement of backbone and sidechain atoms required for guanosine binding and could instead display a preference for other RNA substrates, or even bind nucleic acids non-specifically, as observed in members of the RNase T2 family [48].

A defined negatively charged patch on the CSEP0064/BEC1054 surface is evident (Fig 3E). This feature contrasts with the widespread occurrence of positively charged patches in RNA-binding proteins, with an average area five times larger than negatively charged ones [49]. In CSEP0064/BEC1054, this region may serve to bind RNA via a counterion, as observed in the interaction of ribosomal protein L11 to rRNA using Mg^2+^ [50], or to repel the ligand and electrostatically orient it towards the proposed binding site, as demonstrated for the negatively charged patch of the Gp2 inhibitor of *Escherichia coli* RNA polymerase [51].

Fungal ribotoxins are another class of proteins related to T1 RNases [52]. Submission of CSEP0064/BEC1054 to the Dali server [53] identified fungal ribotoxins as structural homologues of this effector candidate. Ribotoxins display elongated loop regions and a stretch of lysine residues (K111-K113) that are critical for interaction with the SRL bulged G motif [54, 55]. Although CSEP0064/BEC1054 does not display comparable loop regions, it may specifically recognise RNA and/or protein features on the host ribosome to protect rRNA from the action of RIPs from the host. Many other plant ribotoxins also lack the triple lysine motif of fungal ribotoxins (including JIP60), and contact the SRL via alternative sequences, namely conserved tyrosine and tryptophan residues that form the “N-glycosidase signature” [56].

An additional observation is that CSEPs that are predicted to possess an RNase-like fold, also show some conservation of specific amino acid residues within the putative RNA-binding site but not elsewhere (S4 Fig). This conservation underscores the potential importance of the role for RALPHs’ RNA-binding for their effector function: effector paralogues may recognise the same host target, and might have diverged under strong evolutionary pressures to escape recognition by surveilling host resistance (R) proteins [7].

Notably, a CSEP with a predicted RNase-like fold (CSEP0372) has been recently recognised as a CSEP with *MLA* avirulence activity (AVR_a13_) in *B*. *graminis* f.sp. *hordei*. Perception of CSEP0372 by the cognate barley nucleotide-binding domain and leucine-rich repeat (NLR) R protein MLA13 results in isolate-specific resistance. Among 17 fungal isolates examined, this *CSEP* gene was found to be present as five allelic variants of which two confer virulence and three confer avirulence to the fungal pathogen. Another avirulence protein reported in this study (AVR_a1_) shows only weak predicted structural similarity with RNAses [9]. More recently, four additional CSEPs with different *MLA* avirulence activities were identified in *B*. *graminis* f.sp. *hordei* (AVR_a7_, AVR_a9_, AVR_a10_ and AVR_a22_). When analysed on the basis of the CSEP0064/BEC1054 X-ray structure resolved in the present work, only AVR_a7_ and AVR_a13_ exhibited significant structural similarity with CSEP0064/BEC1054, while no meaningful structural similarities with CSEP0064/BEC1054 were noted in the case of AVR_a1_, AVR_a9_, AVR_a10_ and AVR_a22_. In summary, these data suggest that allelic MLA immune receptors are capable to mount potent immune responses upon recognition of structurally unrelated CSEPs as avirulence determinants [57].

### CSEP0064/BEC1054 interferes with induced rRNA cleavage

The structural similarity of CSEP0064/BEC1054 with RNases and ribotoxins raises the possibility that RALPHs like CSEP0064/BEC1054 bind motifs similar to those recognised by RIPs expressed by the plant host. This prompted us to test the effect of CSEP0064/BEC1054 expression on the integrity of host RNAs. In general, MeJA can induce production of RIPs [13], which cleave an adenine base from the large rRNA subunit sugar-phosphate backbone [58–60]. This exposes the phosphodiester bond in the sugar-phosphate backbone, which can then undergo chemical hydrolysis within the cell [58, 61, 62]. *In vitro*, the process can be reconstituted by treatment of depurinated rRNA with aniline, which cleaves the sugar-phosphate backbone at the site of the modified nucleotide [63]. This results in the formation of two defined rRNA fragments, ca. 3,000 and 400 nucleotides long, respectively. The smaller rRNA fragment has been observed in barley and is an indicator of RIP activity [33]. Our experiments showed that RNA extracted from MeJA-induced wheat leaves, treated with aniline, contains a new fragment with an apparent size of about 1,200 nucleotides (Fig 6A). This new RNA species is likely to be a product of depurination/cleavage from a large, abundant RNA, such as rRNA, because it only appears after *in vitro* treatment with aniline. At present we do not know its exact identity, but the size of the RNA fragment is consistent with the products of RNA cleavage previously observed in MeJA-induced RIP, such as JIP60 [33]. The area under the peak was significantly reduced in plants infected with *B*. *graminis* f.sp. *tritici* (Fig 6B). Furthermore, we observed no additional peaks in RNAs from leaves infected by *B*. *graminis* f.sp. *tritici*. The expression of a single transgenic RALPH effector was not as potent as actual infection, but resulted in an intermediate effect which was not statistically different from either the uninfected or infected (azygous) samples. Moreover, the combination of infection and transgenic expression of CSEP0064/BEC1054 was similarly intermediate. It remains to be seen whether expression of multiple RALPH effectors, observed during infection [34], would result in an inhibition that is statistically significant.

### Is CSEP0064/BEC1054 an inhibitor of host RIPs?

We have observed an increased susceptibility in different plants to filamentous pathogens upon expression of CSEP0064/BEC1054 (Fig 1). This effect is consistent with the activities displayed by secreted effectors dedicated to modulate or inhibit the immune system of the host [64]. Although the precise activity of CSEP0064/BEC1054 has not yet been determined, the observations made so far suggest an RNA-binding function that counteracts the role of endogenous plant RNases like RIPs. Depurination of a specific nucleotide in the SRL RNA by RIPs [65] is a mechanism in plants to limit the spread of fungal infections: it impairs binding of the eEF2/GTP complex to the ribosome [66]. This, in turn, inhibits protein synthesis and ultimately leads to apoptosis [67, 68]. Thus, suppressing the function of RIPs could be a prime target for CSEP0064/BEC1054 and other proteins in the large family of RALPHs encoded in the *B*. *graminis* genomes. Several lines of experimental evidence are consistent with this hypothesis. Firstly, our sequence and structural analyses show that this protein is a non-catalytic homolog of fungal RNases (Fig 3C). Secondly, our binding data demonstrate that CSEP0064/BEC1054 is capable of recognising host RNA (Figs 4 and 5A). While the interaction measured is weak, we cannot rule out that the binding affinity of CSEP0064/BEC1054 *in vivo* is enhanced by extended contacts with an RNA motif like the SRL and neighbouring proteins on the ribosomal surface. A similar binding mode has been invoked for ribotoxins and RIPs. For example, structural studies show that the fungal ribotoxin restrictocin can form a stable interaction in solution with the SRL RNA [69], and docking of the restrictocin-SRL RNA complex into the structure of the ribosome showed proximity of this enzyme to ribosomal proteins L6 and L14 [55]. Thirdly, our data also show that CSEP0064/BEC1054 interacts *in planta* with PR10 protein (Fig 2B and 2C). At least some PR10 protein variants have been shown to function as RNase [45] CSEP0064/BEC1054 may thus interfere with barley PR10 RNase function.

In this work we found that the presence of a single CSEP0064/BEC1054 *in planta* appears to afford some protection of a major host RNA species from MeJA-induced cleavage (Fig 6A), but this effect is weak and barely significant on its own. It may be that the expression of several RALPHs are needed to obtain a clear and significant impact. The data is consistent with a model in which CSEP0064/BEC1054 competes with a RIP like JIP60 for an RNA-binding site whose integrity is critical for ribosomal function. This would then meet the need of an obligate biotrophic pathogen, such as the powdery mildew fungus, to deal with the consequences of MeJA induction at early stages of infection [70]. Given the predicted structural similarity of CSEP0064/BEC1054 with more than 100 paralogs in the *B*. *graminis* f.sp. *hordei* CSEP repository [4], it can be expected that additional effector candidates of this pathogen play a similar role. The predicted functional redundancy of these CSEPs might be explained by buffering against the loss of individual effector genes in the highly plastic fungal genome and/or the potential sequential delivery of effector variants during pathogenesis to escape detection by the plant immune system [71].

### Conclusions

**Fig 7:**
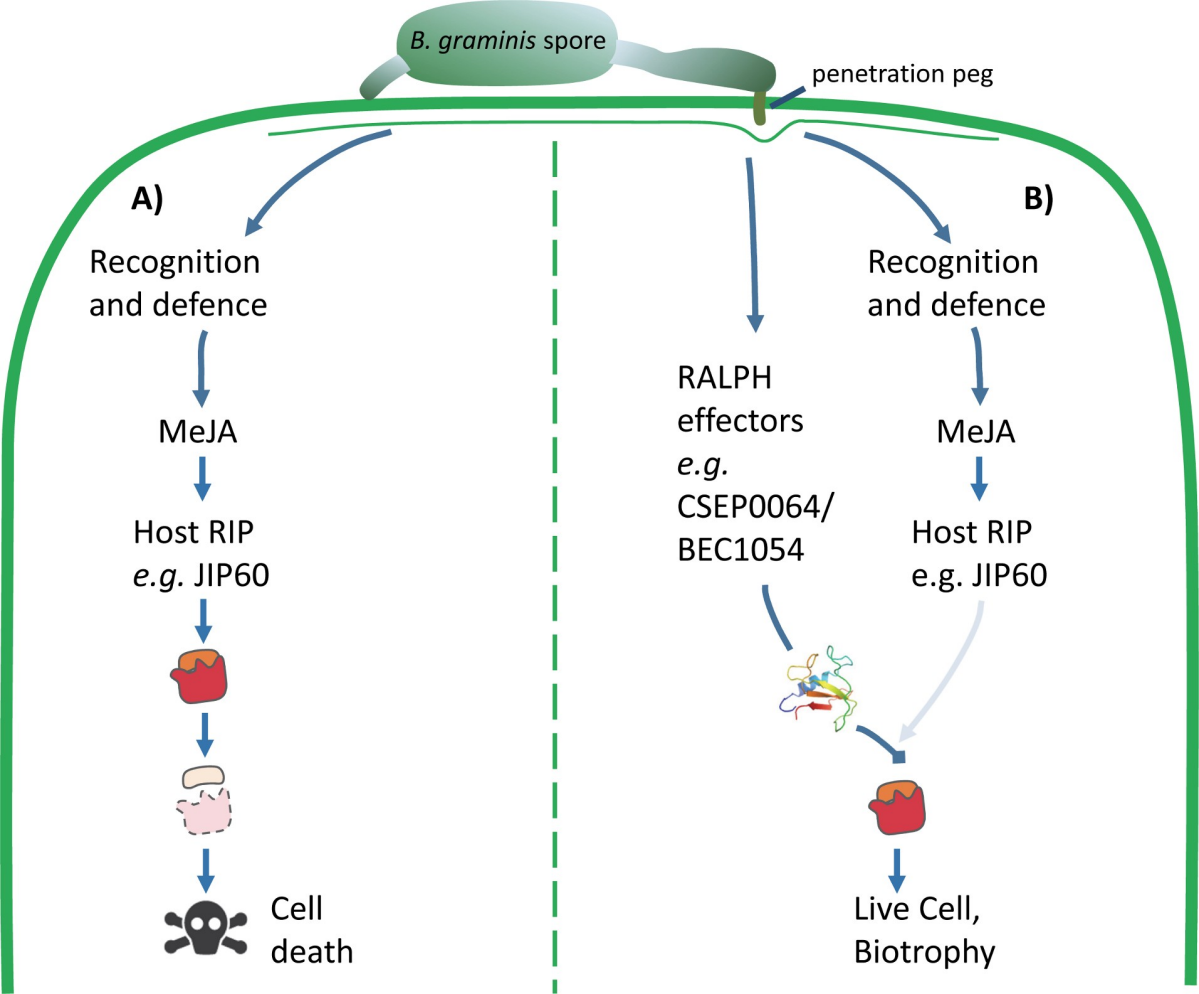
Model of the proposed function of RALPH effectors in the interaction between powdery mildews and host plants. **A)** Plants recognise potential pathogenic microbes by inducing defence responses, such as the production of the jasmonate-induced protein (JIP60) mediated by methyl jasmonate (MeJA). JIP60 is a ribosome-inhibiting protein (RIP) that degrades host ribosomes and triggers cell death. Host cell death is lethal for obligate biotrophic pathogens such as *B*. *graminis*. **B)** Powdery mildew fungi secrete abundant RNase-like proteins from their haustoria (RALPH effectors), such as CSEP0064/BEC1054. We hypothesise that RALPHs facilitate maintenance of live host cells by competing with the host suicide mediated by RIPs such as JIP60.

We hypothesise that CSEP0064/BEC1054 could protect rRNA from the activity of plant RIPs (Fig 7). Preventing the degradation of rRNA would help to preserve the living cell as a food source for the fungus. This is an essential role for a fungus that is an obligate biotrophic pathogen of plants and goes some way to explain why these candidate effectors are such a prominent component of the CSEP complement in grass powdery mildew fungi.

## Methods

### Plant and filamentous pathogen growth conditions

Barley, *H*. *vulgare* L. cv. Golden Promise, and wheat, *T*. *aestivum* L. cv. Cerco, were grown in Levington Seed and Modular Compost Plus Sand FS2 (Everris, Ipswich, UK) in 13 cm square pots. Plants were kept under long-day conditions, 8 h/16 h hours dark/light cycles, at 21 ^o^C and 33% humidity. Seven days after planting, the barley and wheat seedlings were transferred to 216 dm^3^ Perspex boxes, and inoculated with *B*. *graminis* f.sp. *hordei* strain DH14 [8], or *B*. *graminis* f.sp. *tritici* strain “Fielder” (Donal O’Sullivan, NIAB, UK) respectively. The Bimolecular Fluorescence Complementation (BiFC) experiments were performed with barley cv. “Margret”, but grown in 10 cm square pots filled with “Einheitserde” compost (Einheitserde, Frondenberg, Germany). Transgenic wheat experiments were performed using *T*. *aestivum* L. cv. Fielder, which is also susceptible to the strain of *B*. *graminis* f.sp. *tritici* used here. *N*. *benthamiana* was grown in Levington Seed and Modular Compost Plus Sand FS2 (Everris, Ipswich, UK), mixed 2:1 with five-millimetre Vermiculite (5 mm diameter; Sinclair, Lonconshire, UK) in 9 cm square pots, with one plant per pot. Plants were grown under long-day conditions, with 8 h darkness and 16 h light, at 25 ^o^C and 33% humidity, and watered twice per week. Four weeks post planting, detached *N*. *benthamiana* leaves were placed on wet blue-roll (VWR, Chicago, USA), and inoculated with *P*. *tabacina* [72] sporangia suspended in autoclaved demineralised water.

### Gene amplification and entry vector construction

Three days post inoculation (dpi) of barley seedlings with *B*. *graminis*, total RNA was extracted using the using the Qiagen RNeasy Mini Kit (Qiagen, Crawley, UK), and the concentration determined using a NanoDrop-1000 spectrophotometer (Thermo Scientific, Wilmington, USA). Complementary DNA (cDNA) synthesis was performed using 3 mg of barley RNA as a template, with the SuperScript Double-Stranded cDNA Synthesis Kit (Invitrogen, CA, USA). *Jasmonate Induced Protein 60* cDNA (*Jip60*) was amplified from this cDNA template, with PCR conditions as previously described [12]. The resulting PCR product was inserted into the entry vector pCR8 (Invitrogen), as described by the manufacturer.

The initial amplification of genes coding for barley proteins to test for interaction with CSEP0064/BEC1054 was performed in the same manner as for *Jip60*. Re-amplification of the plant genes and fungal genes from the pCR8 vector was performed with Phusion High-Fidelity DNA Polymerase (Thermo Fisher Scientific, Schwerte, Germany). The PCR products were either purified using the DNA Clean & Concentrator TM-5 kit (Zymo Research, Freiburg Germany), or using the QIAquick PCR Purification Kit (Qiagen). The PCR products for pCR8 were treated with restriction enzyme *Dpn*1 (New England Biolabs, Herts, UK) to digest the template plasmid. The remainder were inserted into pDONR201 (Invitrogen, Carlsbad, CA) using the Gateway BP Clonase Enzyme Mix (Invitrogen). The resulting entry vectors were transformed into chemically competent TOP10 *E*. *coli* (Invitrogen), and the transformed bacteria selected on Lysogeny Broth (LB) agar plates containing 100 μg/ml spectinomycin (pCR8) or 50 μg/ml kanamycin (pDONR201). Single colonies from the resulting transformants were picked and grown overnight in LB media with the appropriate antibiotic. Their plasmid DNAs were then purified and sequenced to confirm insertion, orientation and absence of unwanted mutations.

### Vectors for expression in barley and *N*. *benthamiana* through bombardment or agroinfiltration

Gateway LR Clonase enzyme mix (Invitrogen) was used to recombine the Gateway entry vectors with the Gateway expression plasmids to produce the plasmids for bombardment or for agroinfiltration. The resulting plasmids were transformed into chemically competent TOP10 *E*. *coli*, and grown overnight on LB medium supplemented with ampicillin. Single colonies of *E*. *coli* were picked, and the respective plasmids sequenced to confirm the insertion, orientation and absence of unwanted mutations.

For BiFC assays, powdery mildew effector and barley candidate interactor genes were expressed in the pE-SPYCE/pUC-SPYNE-Gateway BiFC vector system [73, 74]. All effectors were expressed in pE-SPYCE-Gateway and tagged N-terminally with the C-terminal half of YFP. All barley genes were expressed in pUC-SPYNE and tagged C-terminally with the N-terminal half of YFP. As transformation control, dsRED expressed in pUbi was used.

For agroinfiltration, *A*. *tumefaciens* (GV3101) was transformed with the plasmids pK7FWG2, pB7RWG2, or pK7WGF2 containing GFP-tagged CSEP0064/BEC1054 [75].

### Transient expression in barley

Bacteria, containing the plasmids to be used for biolistic transformation of barley primary leaves, were cultured overnight in 200 μl LB media with ampicillin, and purified using the NucleoBond Xtra Midi kit (Machery-Nagel, Düren, Germany). Gold micro-carriers (Gold powder, spherical, APS 0.8–1.5 μm, 99.96 (Alfa Aesar, Karlsruhe, Germany), were prepared previously described [76]. In summary, they were weighed in 30 mg aliquots, coated with 5 μl of DNA solution at 100 ng/μl (one μl of DsRED plasmid, 2 μl of bait plasmid and 2 μl of prey plasmid; or for the positive expression controls, 1 μl of DsRED plasmid and 2 μl of expression control plasmid, where all DNA solutions were prepared at 100 ng/μl) whilst being vortexed. Following this, 20 μl of 0.1 M spermidine (Sigma-Aldrich, Munich, Germany) and 50 μl of 2.5 M CaCl_2_ were added dropwise whilst still vortexing. The coated micro-carriers were stored in 60 μl of 100% ethanol on ice until use.

Primary leaves were harvested from seven-day-old barley leaves, and their adaxial surfaces bombarded with coated micro-carriers using a PDS-1000/He System with a Hepta Adaptor as per the manufacturer’s instructions (Bio-Rad, Munich, Germany). A vacuum of 27 inches of mercury was used with 900 psi rupture disks (Bio-Rad).

Following bombardment, barley leaves were maintained on water agar (1% supplemented with 85 μM benzimidazole. Three days after bombardment, the leaves were coated with perfluorodecalin (95%, Sigma-Aldrich) for a minimum of 30 min before imaging. Leaf samples were analysed using a Leica TCS SP8-X laser-scanning microscope (Leica, Wetzlar, Germany), mounted with a 20x 0.75 numerical aperture water-immersion objective. Transformed cells were identified through observation of DsRED fluorescence, which was excited at 561 nm using the 560 nm diode laser. Fluorescence emission was detected between 600 and 640 nm using a photomultiplier tube detector. mYFP fluorescence was excited at 514 nm with an argon laser at 20% base power and 5% laser power for imaging. Fluorescence emission was detected between 520 and 540 nm using a HYD detector with 250 V gain for all images. Sequential imaging was conducted by 400 Hz scanning speed and 5x frame averaging. Image capture and analysis was performed using Leica SP8 software. Further editing was performed using FIJI software v2.0 [77].

For semiquantitative assessment of BiFC signals, transformed cells were manually assigned to one of four categories: strong, medium weak or no detectable YFP fluorescence. Based on 5–8 exemplary cells per category, the following scores were assigned: strong fluorescence (~45% positive pixels per cell– 9), medium fluorescence (~17% positive pixels per cell– 3), weak fluorescence (~5% positive pixels per cell– 1) and no fluorescence (~2% positive pixels per cell– 0). Scoring was done in a blinded manner (identity of samples hidden).

### Agroinfiltration and transient expression in *N*. *benthamiana*

*A*. *tumefaciens* (GV3101) was transformed with the plasmids by electroporation using a MicroPulser Electroporator as per the electroporator manual (BioRad), followed by recovery for two hours in LB media (1 ml), shaking at 28 ^o^C for 2 h. The transformed *Agrobacterium* was subsequently plated onto LB media containing 100 μg of spectinomycin for the plasmids pK7FWG2, pB7RWG2, or pK7WGF2; or 50 μg/ml ampicillin for the colocalisation vectors. Transformed colonies were grown for two days, a single *Agrobacterium* colony selected, the presence of the insert checked by PCR, and the colony streaked onto a fresh plate. Following a further two days of growth, the colonies were resuspended in 2 ml of MMA buffer (10 μM MgCl_2_ and 10 μM MES (2-[N-morpholino] ethanesulfonic acid, pH 5.7). The bacteria were centrifuged (5 min, 8000 g), and resuspended in 2 ml MMA buffer (10 mM). The OD600 was measured, and a bacterial suspension created with a final OD_600_ = 0.5 for RFP constructs, or OD_600_ = 0.2 for GFP constructs.

Three to four weeks old *N*. *benthamiana* plants were selected for agroinfiltration at OD_600_ = 0.5. The *Agrobacterium* suspensions were infiltrated into the abaxial surface of leaves (leaves three and/or four from the apex). The leaves were harvested from the plant two to four days after infiltration, and maintained on damp absorbent paper in clear plastic boxes, under long day conditions (16 h/8 h light/dark photoperiod at 18 ^o^C). Infiltrated leaves were mounted in water, and analysed using a Leica SP5 resonant inverted confocal microscope. Excitation and emission wavelengths were 543 nm and 588 nm, respectively, for RFP, 488 nm and 680 nm for plastid autofluorescence, and 488 nm and 495 nm for GFP. RFP was excited with an argon laser, and autofluorescence and GFP were excited using a helium-neon laser. Image analysis and processing were performed using Leica LAS X (Leica Microsystems, Milton Keynes, UK) and Fiji software (ImageJ).

### *N*. *benthamiana* infection assays

Agrobacteria containing the plasmid pK7FWG2/BEC1054 were infiltrated into one half of an *N*. *benthamiana* leaf, and Agrobacteria with the GFP-only construct into the other. Both *Agrobacterium* strains were infiltrated at an OD_600_ = 0.5. *P*. *tabacina* [72] was used to inoculate the leaves within two hours of agroinfiltration. At ten days after inoculation, inoculated leaves were shaken in water (5 ml) and the number of sporangia harvested were measured through counting with a haemocytometer.

### Generation of transgenic wheat lines

*Wobble CSEP0064/BEC1054* (*wbec1054*) is a synthetic gene corresponding to *B*. *graminis* f.sp. *hordei CSEP0064/BEC1054*, but lacking the N-terminal signal peptide and containing silent “wobble” mutations, minimising the sequence identity of the gene with the wild-type *Blumeria* gene; at the same time, the codon usage was optimised for expression in wheat and barley [11]. The *wbec1054* gene was cloned into pENTRY (Invitrogen) between the attL1 and attL2 sites, and then recombined into the vector pActR1R2-SCV through LR recombination (Invitrogen). such that the *wbec1054* gene would be expressed from the actin promoter *in vivo* [78]. The resulting constructs were transformed into electrocompetent *Agrobacterum* strain AgI1 [79].

The *wbec1054* gene was transformed into *T*. *aestivum* cv. Fielder through *Agrobacterium*-mediated transformation. Immature seeds were collected at 16–20 days post anthesis, and surface-sterilised [80]. Isolated embryos were co-cultivated with *Agrobacterium* at 23°C for two days in the dark [81]. The embryonic axes were removed, and the subsequent tissue culture performed as described previously [80]. The copy number of the *nptII* selectable marker gene was analysed via quantitative real-time PCR (qPCR) using the ΔΔCt method [82]. Plants were grown to maturity, and the T1 generation seeds harvested for further analysis.

### Homozygous line development, genotyping and expression confirmation

Seed dormancy was interrupted by incubation for five days at 32 ^o^C (day), followed by 4 ^o^C for one night. Transgenic wheat seeds were sown in 2x2 cm propagation tray chambers, under the growth conditions listed above for wheat and barley, but with the addition of 2 g/l Osmocote Patterned Release Fertiliser (Everris, Ipswich, UK). Seedlings were transferred after two weeks to square plastic pots (9 cm) containing the same potting mixture.

Transgenic wheat DNA (generations T1 to T3) was extracted using the KAPA3G Plant direct PCR protocol (Kapa Biosystems, ROCHE). Transgene presence and copy number was determined for the generations T1 to T4 by qPCR. Analysis was performed through the ΔΔCt method (ΔΔCt = ΔCt _(control gene)_−ΔCt _(gene of interest)_), with wheat β-tubulin (*tubb6*; U76897.1) as the control, and *wbec1054* as the gene of interest. An expression of 0.00 relative to *tubb6* corresponded to homozygous null plants, referred to hereafter as azygous (-/-), 0.2–0.5 as heterozygous plants (+/-), and 0.5–1 as homozygous plants (+/+).

### Phenotyping transgenic wheat lines

The phenotypic characteristics of mature wheat plants from the T4 generation of lines homozygous (+/+) or azygous (-/-) for *wbec1054* were investigated. Eleven characteristics were assayed: leaf number, maximum height, peduncle (internode 1) and other internode lengths, ear length, subcrown length, fertile tiller number, tiller mass and grain number. Statistical analyses were performed for all characteristics except the subcrown, as the majority of the tiller subcrowns became detached during the drying out phase. The subcrown belonging to the primary tiller could not therefore always be accurately determined.

### Powdery mildew infection assay

Primary leaves were harvested from transgenic wheat plants from the T4 and T5 generations of plants homozygous or azygous for *wbec1054*, and segments (2 cm each) taken from the base, middle and tip using a flat blade. The “mature” leaves correspond to leaf four on eleven-week-old plants, “young” leaves were the primary leaves from three week-old plants. The leaf segments were placed on wet blue-roll paper, and inoculated with *B*. *graminis* f.sp. *tritici* (isolate “Fielder”) on the adaxial leaf surface. One hour post inoculation, leaf segments were transferred onto water agar (0.5% agar supplemented with 16 mg/l benzimidazole) with the infected adaxial side up. Plates were maintained for three days under the growing conditions described above (Plant and filamentous pathogen growth conditions).

Staining of infected leaf segments was performed using 0.1% trypan blue dye in ethanolic lactophenol (1:3.35) (RAL Diagnostics, Martillac, France) for two hours at 80 ^o^C. Destaining was then performed using chloral hydrate (2 mg/ml) for 2 h. A Carl Zeiss Axioskop 2 plus microscope (Zeiss, Cambridge, UK) was used to view fungal structures. The proportion of germinated conidia that formed at least one haustorium (propH) was calculated (where propH = (haustorial forming germinated conidia/total number of germinated conidia)). Colonies that formed epiphytic hyphae were used as a proxy for the presence of at least one functional haustorium.

### RNA extraction and analysis

Total RNA was extracted using the Qiagen RNeasy Mini Kit (Qiagen). RNA was quantified with a NanoDrop-1000 spectrophotometer (Thermo Scientific). Aniline (1.2 μl, 1 M)(≥99.5%, Sigma) was used to treat 10 μl, containing up to 1 μg of extracted RNA in RNase free water, and incubated in the dark at 60 ^o^C for three minutes, following which 2 μl of 5 M ammonium acetate stop solution with 100 mM EDTA was added and the mixture transferred on ice. The RNA was then precipitated by adding ethanol (1 ml), incubated at -80 ^o^C for 20 min, and then collected by centrifugation. The quantity of RNA recovered was measured (NanoDrop 1000) and then analysed using an Bioanalyzer RNA Nano 6000 kit (Bioanalyzer 2100, Agilent Technologies, Santa Clara, CA). The peaks of interest were indentified manually, and the areas under the peaks measured using the manucfacturer’s software (Agilent Technologies 2100 Expert, 2009). The order of the cytoplasmic and chloroplastic rRNA peaks was obtained from the manufacturer’s guide book (http://citeseerx.ist.psu.edu/viewdoc/download?doi=10.1.1.493.5004&rep=rep1&type=pdf). The sizes of the small and large peaks were obtained from the following PDB models DOI: 10.2210/pdb4v7e/pdb and DOI: 10.2210/pdb5mmj/pdb.

### Production of recombinant CSEP0064/BEC1054 for crystallisation

A gene fusion coding for thioredoxin, a hexahistidine tag, a TEV digestion site and the mature form of CSEP0064/BEC1054 (Uniprot N1JJ94, residues 22–118) was expressed in the pNIC-Trx plasmid (kindly provided by Oxford SGC) using the Shuffle T7 Express (NEB) *E*. *coli* strain. Cell pellets were resuspended in 50 mM Tris, 300 mM NaCl, pH 8.0 (buffer A), and lysed at 25 kpsi using a cell disruptor (Constant Systems Ltd, Warwickshire, UK). Post clarification, supernatants were loaded onto Ni-NTA resin (Qiagen), and washed with buffer A containing 10 mM imidazole prior to elution with buffer A containing 300 mM imidazole. After dialysis in buffer A, CSEP0064/BEC1054 was digested with TEV protease in the same buffer and passed down Ni-NTA resin as described previously to remove thioredoxin and the hexahisitidine tag, prior to size exclusion chromatography on an S75 16/60 column (GE Healthcare, Buckinghamshire, UK) in 20 mM phosphate pH 7.0, 150 mM NaCl (buffer B).

### Crystallisation of CSEP0064/BEC1054 and structure solution

Purified CSEP0064/BEC1054 was dialysed into crystallisation buffer (10 mM Tris, 150 mM NaCl, pH 7.0) and concentrated to 10 mg/ml for crystallisation. A variety of commercially available solution conditions for crystallisation (Hampton Research,CA, USA) were screened using the sitting-drop vapour diffusion method. CSEP0064/BEC1054 was combined with the mother liquor on a 1:1 ratio in 200 nl drops. Crystals obtained in 0.1 M sodium acetate buffer pH 5.0, supplemented with 30% PEG 4000, 0.4 M (NH_4_)_2_SO_4_ were cryoprotected with 30% glycerol and flash frozen for data collection. A native dataset to 1.3 Å resolution was collected at a wavelength of 0.92 Å using the I04 beamline (DIAMOND Light Source, Oxford, UK) and a second derivative dataset collected at a wavelength of 0.95 Å (above the iodide f” edge) from crystals that had been soaked in mother liquor supplemented with 0.5 M sodium iodide. Initial processing, scaling and structure factor calculation of native and iodide SAD datasets was performed using XDS [83, 84] and TRUNCATE [85] respectively, from within the XIA2 program [86]. Phasing of the derivative dataset was performed via single-wavelength anomalous diffraction with density modification, using autoSHARP software (Global Phasing Ltd., Cambridgeshire, UK) [87]. Following calculation of protein phases, a partial model was built automatically in ARP/wARP [88]. The model was extended through manual model-building in Coot [89].

The refined CSEP0064/BEC1054 structure was thus used as a search model to phase the higher resolution native dataset via molecular replacement using Phaser MR [90]. Iterative cycles of model building and reciprocal space refinement were performed in Coot [89] and Refmac5 [91], respectively, until convergence of R_work_ values, where 5% of reflections were excluded for cross-validation.

Both models obtained from SAD and native datasets contained a single copy of CSEP0064/BEC1054 in the asymmetric unit, and all residues were built with the exception of the N-terminal alanine, the side chain of R76 and the C-terminal G97, due to missing or ambiguous density in maps. Model validation was performed using tools in Molprobity (Chen, Arendell et al., 2010).

### CSEP0064/BEC1054 backbone assignment

CSEP0064/BEC1054 was concentrated to 300 μM in buffer B for NMR experiments, where 10% ^2^H_2_O v/v was added to provide a deuterium lock signal. 2D ^1^H^15^N HSQC spectra (Kay et al., 1992, 1994) were recorded at 308 K on a Bruker 600 MHz AvanceIII spectrometer equipped with a TCI cryoprobe (Cross Faculty Centre for NMR, Imperial College London, UK). The chemical shifts of the Cα, Cβ, HN, and CO atoms of the ^13^C, ^15^N labelled CSEP0064/BEC1054 protein were obtained from HNCACB/CBCA(CO)NH and HNCO/HN(CA)CO experiments using standard methods. Data were processed using NMR-Pipe [92], and analysed in CCPN Analysis [93] or using an in-house version of NMRView (One Moon Scientific) [94].

### CSEP0064/BEC1054 RNA binding assays

For MST experiments, the recombinant CSEP0064/BEC1054 protein was labelled using the Monolith NT.115 protein labelling kit (NanoTemper technologies, Munich Germany) using red fluorescent dye NT-647 NHS (amine-reactive) according to the manufacturer’s instructions. Assays were performed using a Monolith NT.115 MST machine (Nanotemper Technologies), where LED power was kept at 20% and MST power at 40%. Assays were performed in standard or hydrophilic capillaries in 20 mM Tris buffer pH 7.4, 150 mM NaCl, 0.05% Tween, where RNA was titrated against labelled CSEP0064/BEC1054 kept at a concentration of 50 nM. CSEP0064/BEC1054 was titrated with total RNA (extracted from barley) from a starting concentration of 10 μg/ul, or *in vitro* transcribed SRL RNA (5´-ACCUGCUCAGUACGAGAGGAACCGCAGGU-3´) or bacteriophage T7 promoter sequences (5´-AATTTAATACGACTCACTATAGG-3´) from a starting concentration of 1 mM. Curve fitting was performed using the NTAnalysis software (Nanotemper Technologies) in the Thermophoresis + T-Jump mode for the SRL ligand, and using the Hill equation for the total RNA. K_D_ values calculated using non-linear regression.

For NMR experiments, CSEP0064/BEC1054 protein at a concentration of 50 μM was titrated with from 0.5 to up to 80 M equivalents of a DNA SRL oligonucleotide sequence, where 2D ^1^H^15^N HSQC spectra were recorded for each titration point as outlined. Experiments were performed in 50 mM Tris, 150 mM NaCl, at 308 K. Analysis of chemical shift perturbations was performed in CCPN Analysis [93].

### Statistical analyses

Bartlett tests were performed for numerical datasets to determine whether the variance was homogeneous [95]. General Linear Models (GLMs) were conducted on all datasets except for the infection of transgenic wheat with *B*. *graminis* f.sp. *tritici*, where a Generalised Linear Mixed Model (GLMM) was utilised. Where possible, model simplification was performed, and non-significant or non-interacting factors removed, resulting in the minimal model. For the GLMs, linear hypotheses were tested in a pairwise manner using Games-Howell *post-hoc* tests. The only assay in which a two-way interaction of factors was detected was the RNA extraction and analysis (described above).

A GLMM was used, as non-normal repeated measures (i.e. the fact that the old and young leaves were from the same plants, and the different leaf sections used originated from the same leaves), can be accounted for through the addition of random effects [95]. For the GLMM, the count data corresponding to the total number of germinated conidia (with and without functional haustoria) was bound as a single vector, creating the response variable “y”. To take into account pseudoreplication (due to sampling repeatedly from the same plants/leaves), age, genotype and leaf segment were set as fixed effects, and a binomial family was used due to the response variable being count data. The linear hypotheses were investigated in a pairwise manner using the “multcomp” package in R.

For the phenotyping assay, a “Poisson” family structure was used to account for count data, and datasets were logged to account for overdispersion in the original models (where the residual deviance is much greater than the degrees of freedom).

### Accession numbers

Sequence data from this article can be found in the EMBL/GenBank data libraries under the following accession number(s): 40S 16, accession KP293844; eEF1γ, accession KP293852; eEF1α(1) and eEF1α(3), accessions KP293845 and KP293846; GST, accession KP293847; MDH, accession KP293848; NDPK, accession KP293849; PR10, accession KP293851; CSEP0064/BEC1054, accession CCU83233.1; CSEP0264, accession CCU83219.1; and CSEP0102, accession CCU74258.1. The structure of CSEP0064/BEC1054 is available in protein data bank (PDB) under accession number 6FMB.

## Supporting information

S1 Fig The phenotype of transgenic wheat is unaffected by the presence of the *wbec1054* transgene, which encodes CSEP0064/BEC1054. The T4 generation of transgenic wheat either homozygous (+/+) or azygous (-/-) for CSEP0064/BEC1054 was grown in a random plot design, and the phenotypic characteristics of adult plants were investigated. The boxes represent the quartiles, the thick line denotes the median, and maximum and minimum values are shown by the error bars, and circles indicate outliers. *Post-hoc* tests indicated that none of the phenotypic characteristics were significantly different under the experimental conditions used. Genotyping and qPCR were used to determine that *wbec1054* was present and transcribed in the homozygous wheat line 3.3.14, and absent in the azygous line 3.3.12. A randomised block design, with six plots, was used [17, 19] with each block representing one wheat plant. All seeds used to grow the plants were of the same age, and had been stored under the same conditions.
(TIF)

S2 Fig CSEP and host proteins used for BiFC analysis are expressed in barley leaf epidermal cells. (A) Representative micrographs showing expression of C-terminally mYFP-tagged CSEP0064/BEC1054, CSEP0264 and CSEP0102 (lacking the N-terminal signal peptide) in single barley leaf epidermal cells. Maximum projections (combined Z-stack) are shown. Scale bars are 20 μM. (B) Representative micrographs showing expression of C-terminally mYFP-tagged PR10, eEF1α(1), eEF1α(3), eEF1γ, 40S 16, MDH, GST and NDPK in single barley leaf epidermal cells. Single focal planes are shown. Scale bars are 20 μm.
(TIF)

S3 Fig ^1^H-^15^N HSQC spectrum showing NMR backbone assignments of CSEP0064/BEC1054. Spectra used to obtain these assignments were recorded in 50 mM sodium phosphate, 150 mM NaCl, pH 7.4 at 308 K and 600 MHz. Residues with doubled amide cross peaks in the ^1^H and ^15^N frequencies (suggesting alternative conformations due to *cis-trans* isomerisation of prolines 12 and 54) are marked with an asterisk. Resonances from side chain amides (upper right) are connected by a straight line.
(TIF)

S4 Fig Sequence conservation between microbial RNases and *Blumeria graminis* f.sp. *hordei* CSEPs predicted to have an RNase fold mapped onto the crystal structure of CSEP0064/BEC1054. Sequence conservation was calculated using the ConSurf server [98, 99], where MSAs were supplied from the ClustalOmega server (Analysis Tool Web Services from the EMBL-EBI.(2013 May 13); Nucleic Acids Research 41 (Web Server issue):W597-600). Highly conserved residues are coloured magenta, through white to cyan for regions of low sequence conservation. Aromatics that are also conserved in the T1 RNase family are indicated.
(TIF)

S5 Fig **Quantification of Bioanalyzer RNA peak areas. A**) An Agilent Bioanalyzer 2100 was used to measure total RNA was run on a Bioanalyzer RNA Nano 6000 chip. The peak areas were calculated using Agilent 2100 Expert software via manual boundary assignment for the peaks of interest. The asterisk symbol “*” for the small diagnostic peak, “16S” for the small chloroplastic rRNA, “18S” for the small cytoplasmic rRNA, “23S” for the large chloroplastic rRNA and “28S” for the large cytoplasmic rRNA, FU” for fluorescence units. Determining the size of the novel RNA peak. An Agilent Bioanalyzer 2100 was used to measure total RNA on a Bioanalyzer RNA Nano 6000 chip. An RNA ladder, consisting of single stranded RNA fragments of known size, was used to estimate the size of peaks within samples of total RNA. The RNA ladder peaks are represented by white circles. The blue cross represents the novel peak, which ran at an approximate size of 1,200 bases. The blue triangles represent the running times and sizes of the chloroplastic and cytoplasmic rRNAs, and are labelled with both their names and sizes, with the abbreviations “16S” for the small chloroplastic rRNA, “18S” for the small cytoplasmic rRNA, “23S” for the large chloroplastic rRNA and “28S” for the large cytoplasmic rRNA.
(TIF)

S6 Fig Transgenic expression of CSEP0064/BEC1054 inhibits specific MeJA-dependent aniline-induced rRNA degradation: Data from additional transgenic line 3.3.7. Electropherogram peak analysis of total RNA from wheat leaves (line 3.3.7). The area of the degradation peak was calculated in relation to the area of the 28S rRNA (28S). Total rRNA was extracted from transgenic wheat plants that were firstly, in case of the infected plants, inoculated with *B*. *graminis* f.sp. *tritici* for three days, and then treated with 40 μM MeJA for the following five days. *Post-hoc* tests were used to determine whether +/+ and -/- plants undergoing the same treatment were significantly different, as is indicated by different letters. The boxes represent the quartiles, the thick line denotes the median, and maximum and minimum values are shown by the error bars.
(TIF)

S1 Table The proportion of conidia that formed at least one haustorium (propH) is affected by the presence of the transgene *wbec1054*, which encodes *Blumeria* Effector Candidate 1054, and by the sampling location. Multiple Comparisons of Means with Tukey Contrasts were conducted to identify whether the mean propH differed for plants homozygous (+/+) or azygous (-/-) for CSEP0064/BEC1054, or for samples taken from the base, middle or tip of the leaf. Significant difference is indicated by “***” for p≤0.005.
(DOCX)

S2 Table Data processing and refinement statistics for the structures of CSEP0064/BEC0054. (DOCX)
