# Supplementary figures and images for: The fungal ribonuclease-like effector protein CSEP0064/BEC1054 represses plant immunity and interferes with degradation of host ribosomal RNA
Source: PLoS Pathog. 2019 Mar 11;15(3):e1007620. doi: 10.1371/journal.ppat.1007620 (PMC6464244; doi:10.1371/journal.ppat.1007620)

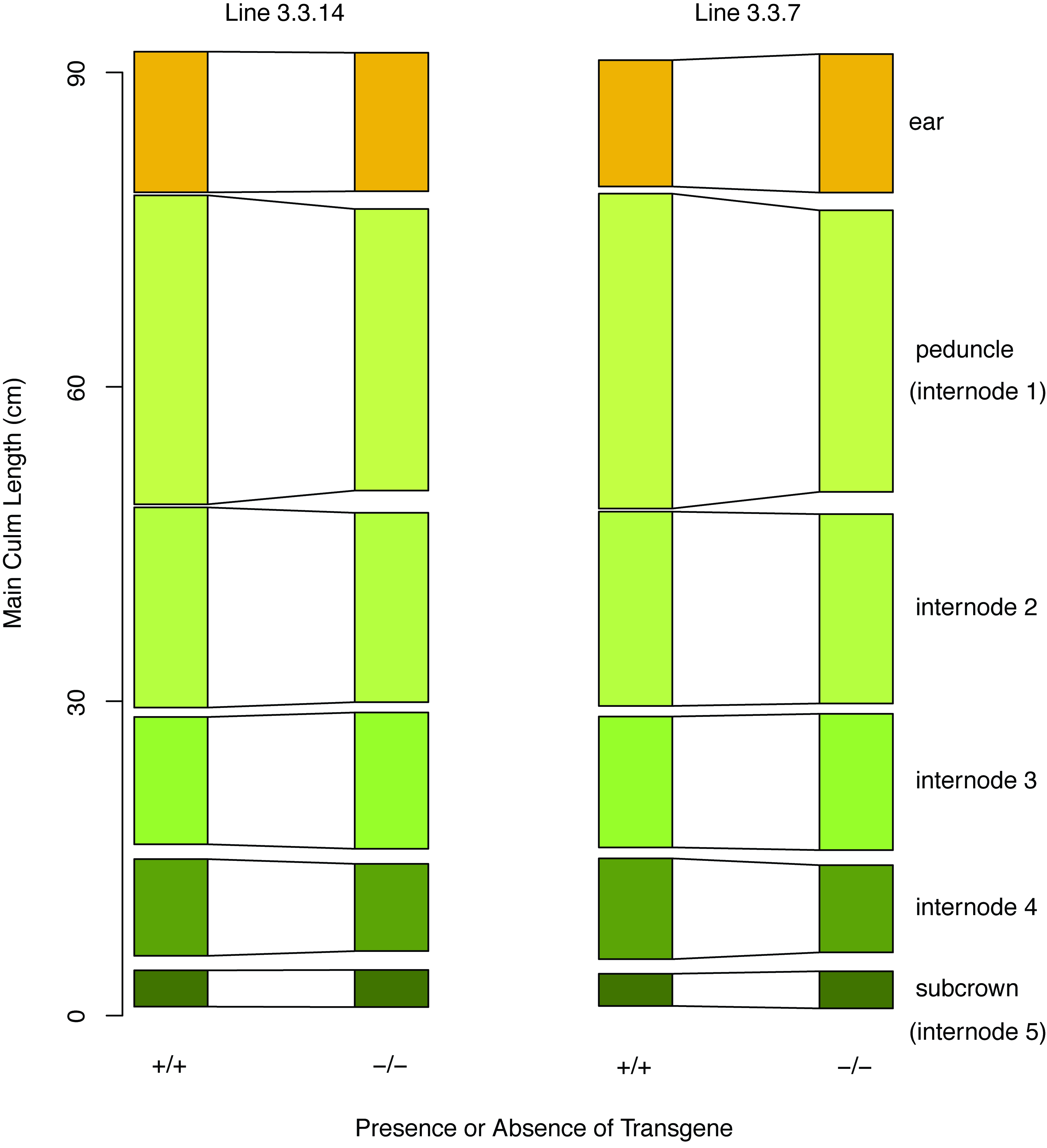

Supplement: S1 Fig — The T4 generation of transgenic wheat either homozygous (+/+) or azygous (-/-) for CSEP0064/BEC1054 was grown in a random plot design, and the phenotypic characteristics of adult plants were investigated. The boxes represent the quartiles, the thick line denotes the median, and maximum and minimum values are shown by the error bars, and circles indicate outliers. Post-hoc tests indicated that none of the phenotypic characteristics were significantly different under the experimental conditions used. Genotyping and qPCR were used to determine that wbec1054 was present and transcribed in the homozygous wheat line 3.3.14, and absent in the azygous line 3.3.12. A randomised block design, with six plots, was used [17, 19] with each block representing one wheat plant. All seeds used to grow the plants were of the same age, and had been stored under the same conditions. (TIF) [file ppat.1007620.s001.tif]

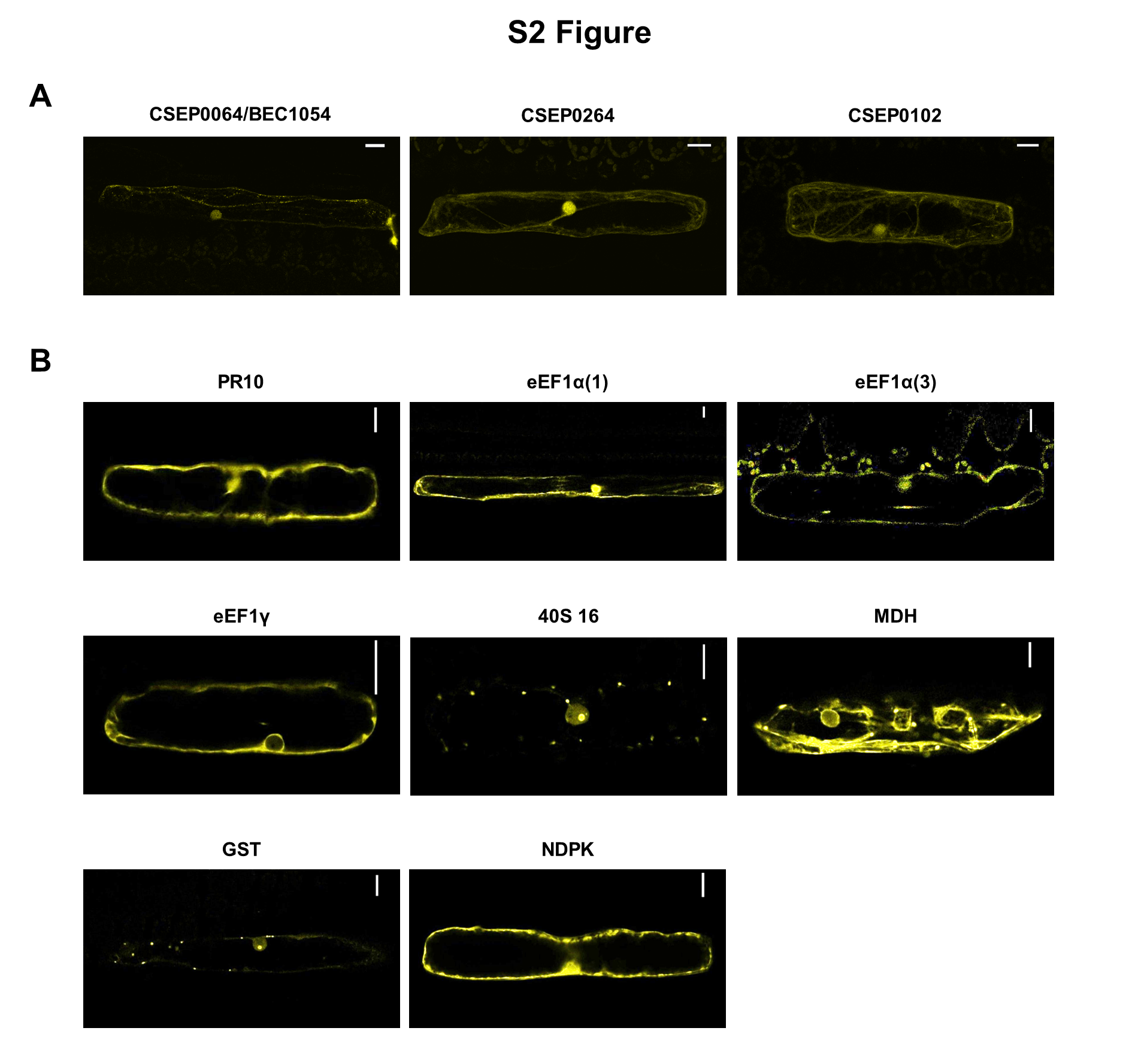

Supplement: S2 Fig — (A) Representative micrographs showing expression of C-terminally mYFP-tagged CSEP0064/BEC1054, CSEP0264 and CSEP0102 (lacking the N-terminal signal peptide) in single barley leaf epidermal cells. Maximum projections (combined Z-stack) are shown. Scale bars are 20 μM. (B) Representative micrographs showing expression of C-terminally mYFP-tagged PR10, eEF1α(1), eEF1α(3), eEF1γ, 40S 16, MDH, GST and NDPK in single barley leaf epidermal cells. Single focal planes are shown. Scale bars are 20 μm. (TIF) [file ppat.1007620.s002.tif]

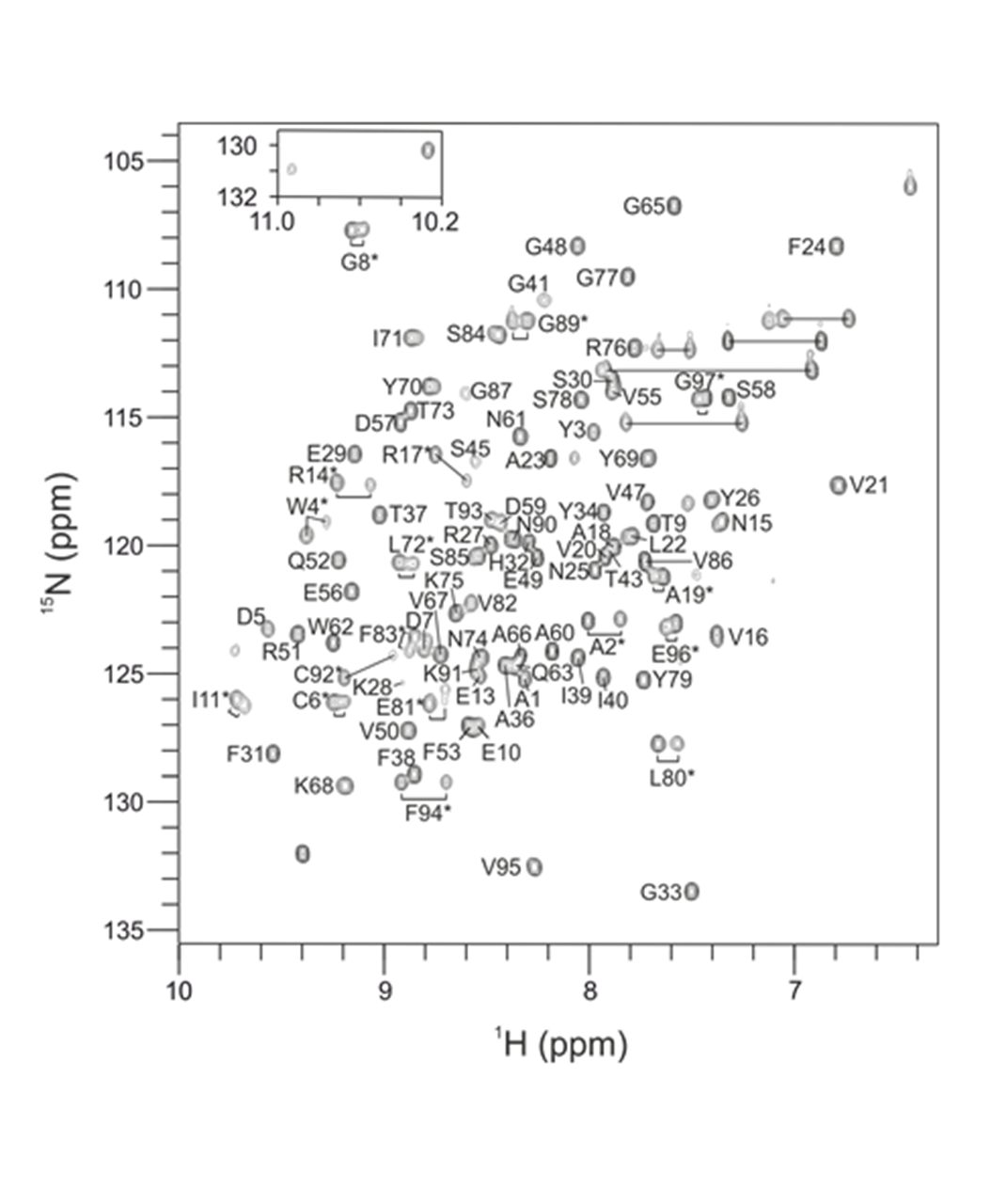

Supplement: S3 Fig — Spectra used to obtain these assignments were recorded in 50 mM sodium phosphate, 150 mM NaCl, pH 7.4 at 308 K and 600 MHz. Residues with doubled amide cross peaks in the 1H and 15N frequencies (suggesting alternative conformations due to cis-trans isomerisation of prolines 12 and 54) are marked with an asterisk. Resonances from side chain amides (upper right) are connected by a straight line. (TIF) [file ppat.1007620.s003.tif]

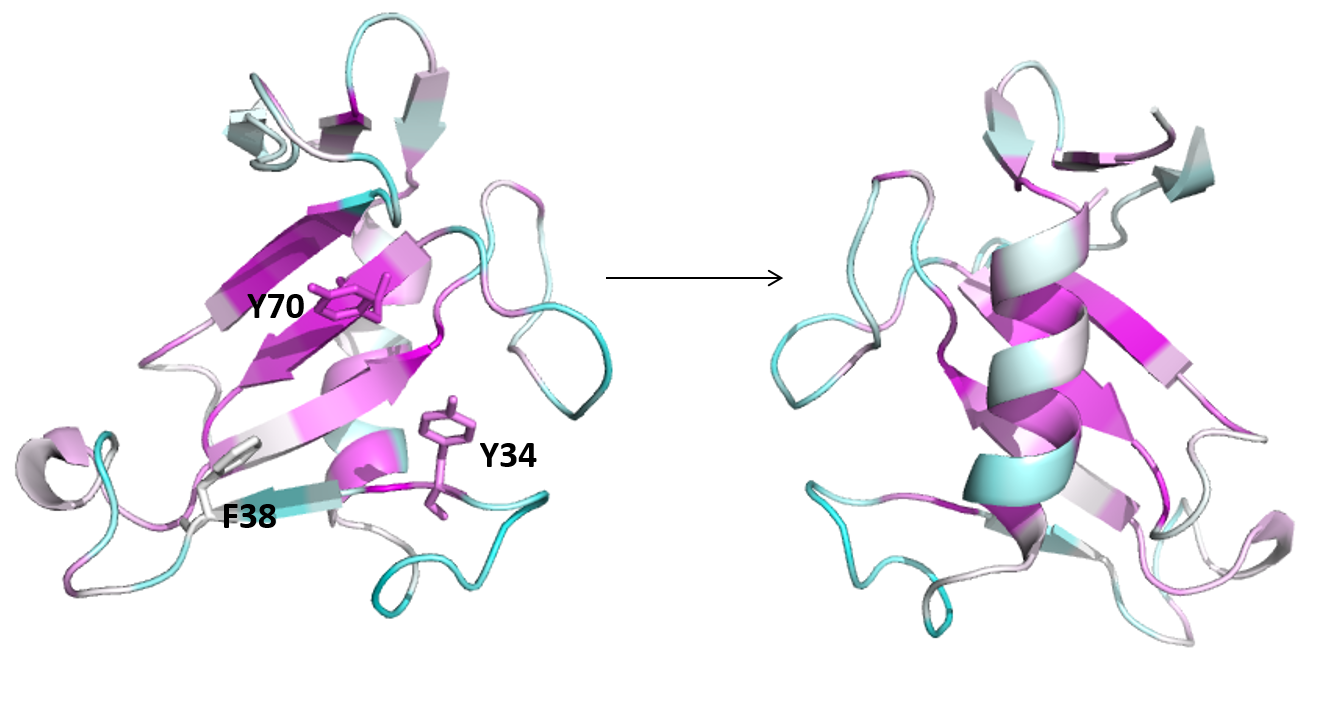

Supplement: S4 Fig — Sequence conservation was calculated using the ConSurf server [98, 99], where MSAs were supplied from the ClustalOmega server (Analysis Tool Web Services from the EMBL-EBI.(2013 May 13); Nucleic Acids Research 41 (Web Server issue):W597-600). Highly conserved residues are coloured magenta, through white to cyan for regions of low sequence conservation. Aromatics that are also conserved in the T1 RNase family are indicated. (TIF) [file ppat.1007620.s004.tif]

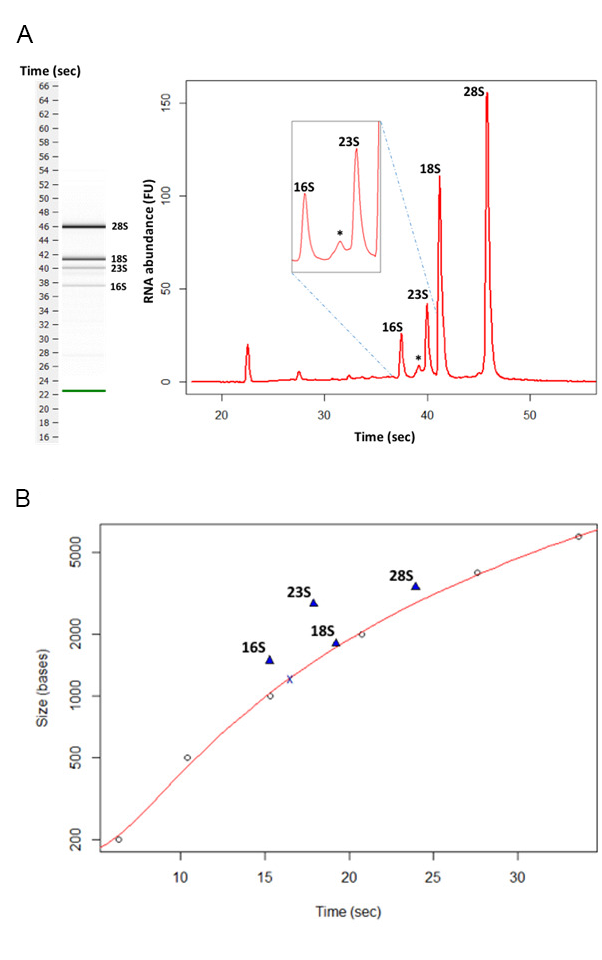

Supplement: S5 Fig — Quantification of Bioanalyzer RNA peak areas. A) An Agilent Bioanalyzer 2100 was used to measure total RNA was run on a Bioanalyzer RNA Nano 6000 chip. The peak areas were calculated using Agilent 2100 Expert software via manual boundary assignment for the peaks of interest. The asterisk symbol “*” for the small diagnostic peak, “16S” for the small chloroplastic rRNA, “18S” for the small cytoplasmic rRNA, “23S” for the large chloroplastic rRNA and “28S” for the large cytoplasmic rRNA, FU” for fluorescence units. Determining the size of the novel RNA peak. An Agilent Bioanalyzer 2100 was used to measure total RNA on a Bioanalyzer RNA Nano 6000 chip. An RNA ladder, consisting of single stranded RNA fragments of known size, was used to estimate the size of peaks within samples of total RNA. The RNA ladder peaks are represented by white circles. The blue cross represents the novel peak, which ran at an approximate size of 1,200 bases. The blue triangles represent the running times and sizes of the chloroplastic and cytoplasmic rRNAs, and are labelled with both their names and sizes, with the abbreviations “16S” for the small chloroplastic rRNA, “18S” for the small cytoplasmic rRNA, “23S” for the large chloroplastic rRNA and “28S” for the large cytoplasmic rRNA. (TIF) [file ppat.1007620.s005.tif]

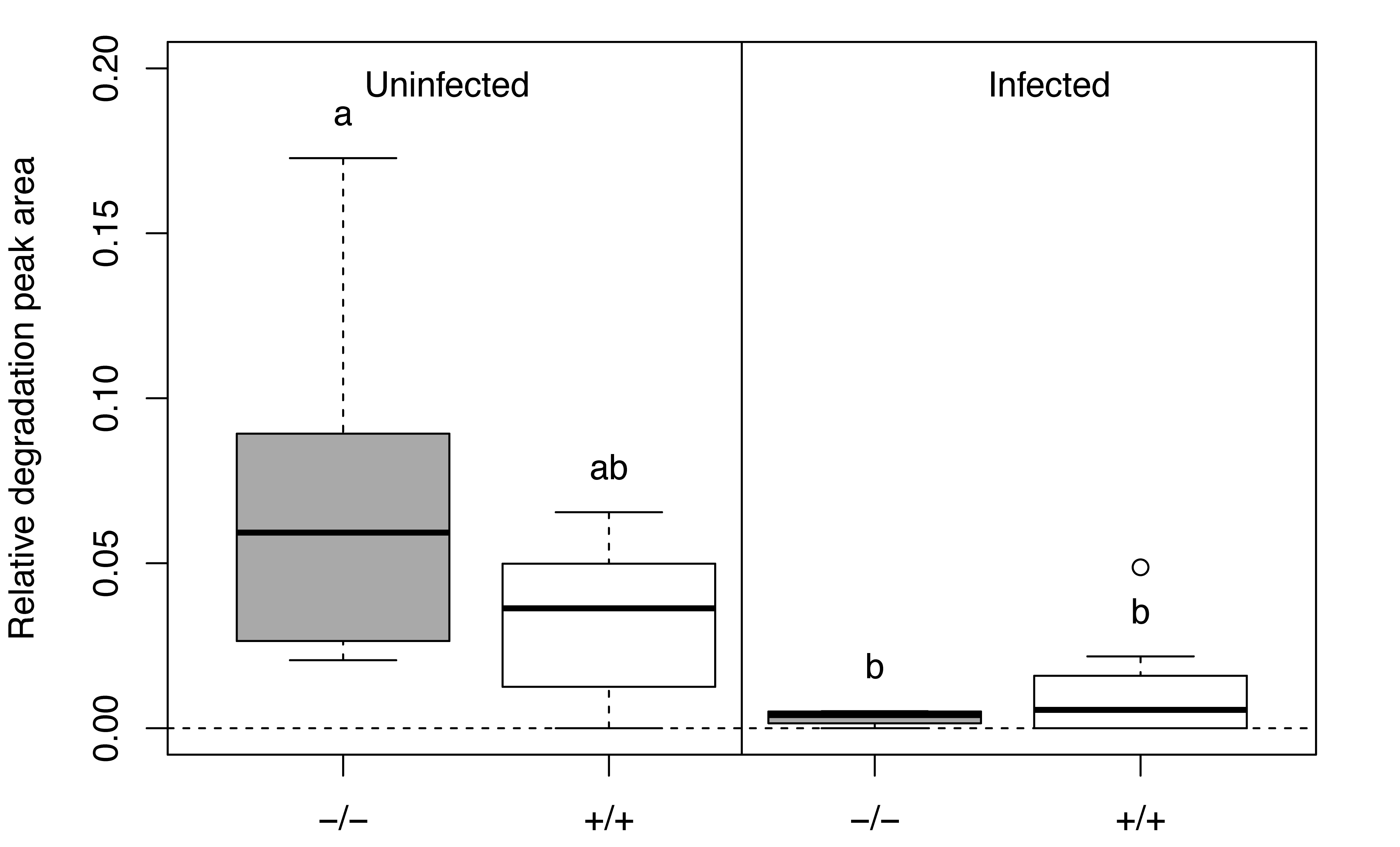

Supplement: S6 Fig — Electropherogram peak analysis of total RNA from wheat leaves (line 3.3.7). The area of the degradation peak was calculated in relation to the area of the 28S rRNA (28S). Total rRNA was extracted from transgenic wheat plants that were firstly, in case of the infected plants, inoculated with B. graminis f.sp. tritici for three days, and then treated with 40 μM MeJA for the following five days. Post-hoc tests were used to determine whether +/+ and -/- plants undergoing the same treatment were significantly different, as is indicated by different letters. The boxes represent the quartiles, the thick line denotes the median, and maximum and minimum values are shown by the error bars. (TIF) [file ppat.1007620.s006.tif]
