# Supplementary material for: The fungal ribonuclease-like effector protein CSEP0064/BEC1054 represses plant immunity and interferes with degradation of host ribosomal RNA
Source: PLoS Pathog. 2019 Mar 11;15(3):e1007620. doi: 10.1371/journal.ppat.1007620 (PMC6464244; doi:10.1371/journal.ppat.1007620)
Supplement: S1 Table — Multiple Comparisons of Means with Tukey Contrasts were conducted to identify whether the mean propH differed for plants homozygous (+/+) or azygous (-/-) for CSEP0064/BEC1054, or for samples taken from the base, middle or tip of the leaf. Significant difference is indicated by “***” for p≤0.005. (DOCX) [file ppat.1007620.s007.docx]

**S1 Table: The proportion of conidia that formed at least one haustorium**

|  |  | Estimate | Standard error | z-value | p-value | Significance |
| --- | --- | --- | --- | --- | --- | --- |
| +/+ | -/- | 0.66 | 0.10 | 6.35 | 2.21e-10 | *** |
| Middle | Base | 0.59 | 0.11 | 5.41 | 6.42e-08 | *** |
| Tip | Base | 1.09 | 0.16 | 6.71 | 1.94e-11 | *** |

The proportion of conidia that formed at least one haustorium (propH) is affected by the presence of the transgene *wbec1054*, which encodes CSEP0064/BEC1054, and by the sampling location. Multiple comparisons of means with Tukey contrasts were conducted to identify whether the mean propH differed for plants homozygous (+/+) or azygous (-/-) for CSEP0064/BEC1054, or for samples taken from the base, middle or tip of the leaf. Significant difference is indicated by “***” for p≤0.001.
