# Supplementary material for: The fungal ribonuclease-like effector protein CSEP0064/BEC1054 represses plant immunity and interferes with degradation of host ribosomal RNA
Source: PLoS Pathog. 2019 Mar 11;15(3):e1007620. doi: 10.1371/journal.ppat.1007620 (PMC6464244; doi:10.1371/journal.ppat.1007620)
Supplement: S2 Table — (DOCX) [file ppat.1007620.s008.docx]

## S2 Table Data processing and refinement statistics for the structures of CSEP0064/BEC1054.

| **Data collection** | **Iodide SAD** | **Native** |
| --- | --- | --- |
| Diffraction source | I04-1, DIAMOND Light Source | I04, DIAMOND Light Source |
| Wavelength (Å) | 0.9200 | 0.9200 |
| Space group | P6_2_ 2 2 | P6_2_ 2 2 |
| Unit cell dimensions (a, b, c) (Å) | 60.04 60.04 77.75 | 60.52 60.52 78.28 |
| Unit cell angles (α, β, γ (^o^) | 90.00 90.00 120.00 | 90.00 90.00 120.00 |
| Resolution range (Å) | 52.00-1.90 (1.95-1.90) | 52.41-1.30 (1.34-1.30) |
| I/σ(I) | 39.7 (11.0) | 23.4 (5.0) |
| R_pim_ | 0.016 (0.072) | 0.017 (0.163) |
| CC_1/2_ | 0.782 (0.541) | 0.998 (0.996) |
| Total number of reflections | 128596 (5333) | 670160 (25064) |
| Total number of unique reflections | 6985 (498) | 21286 (1483) |
| Completeness (%) | 100.0 (99.8) | 99.5 (96.5) |
| Multiplicity | 18.4 (10.7) | 31.5 (16.9) |
| CC_1/2_ anom | 0.782 (0.541) | - |
| I/ σ(I)anom | 1.95 (1.01) | - |
| Anomalous completeness (%) | 99.9 (99.0) | - |
| Anomalous multiplicity | 10.2 (5.70) | - |
| **Refinement** |  |  |
| No of reflections Working | 6626 | 20158 |
| % Free | 4.77 | 5.15 |
| R_work_ | 0.1475 | 0.1437 |
| R_free_ | 0.1975 | 0.1764 |
| **Structure/Stereochemistry** |  |  |
| Number of atoms protein | 760 | 758 |
| solvent | 89 | 86 |
| ligand | 3 (Iodides) | - |
| r.m.s.d. bond lengths (Å) | 0.0223 | 0.0109 |
| r.m.s.d. bond angles (^o^) | 2.0057 | 1.5684 |
| PDB ID |  |  |
